# Siderophores and secondary metabolites produced by Ganoderma adspersum

**DOI:** 10.1099/mic.0.001621

**Published:** 2025-11-14

**Authors:** Carolina Reyes, Steven Ahrendt, Robert Riley, Anna Lipzen, Vivian Ng, Igor V. Grigoriev, Francis W. M. R. Schwarze, Oliver Baars

**Affiliations:** 1 Laboratory for Cellulose and Wood Materials, Empa, Überlandstrasse 129, 8600 Dübendorf, Switzerland; 2 U.S. Department of Energy Joint Genome Institute, Lawrence Berkeley National Laboratory, Berkeley, CA 94720, USA; 3 Department of Plant and Microbial Biology, University of California, Berkeley, CA 94720, USA; 4 Laboratory for Cellulose and Wood Materials, Empa, Lerchenfeldstrasse 5, 9014 St. Gallen, Switzerland; 5 Department of Entomology and Plant Pathology, North Carolina State University, 840 Main Campus Drive, Raleigh, NC, 27695, USA

**Keywords:** fungal-genome, *Ganoderma*, iron, Siderophores, terpenes

## Abstract

*Ganoderma adspersum* is a white-rot wood-degrading basidiomycete of ecological, biotechnological and medicinal interest. In addition to its role in lignin degradation, it produces bioactive metabolites with reported antimicrobial and antioxidant activities. However, the mechanisms of iron acquisition, including siderophore-mediated pathways, remain poorly characterized in *Ganoderma* species. Improved understanding of these systems is essential to elucidate their contributions to fungal physiology, secondary metabolism and ecological adaptation. In this study, the genome of *G. adspersum* was sequenced for the first time and screened for genes that may be involved in the production of secondary metabolites. A gene cluster was identified as potentially involved in iron uptake. In particular, genes related to non-ribosomal peptide synthetases were detected next to a gene encoding a monooxygenase and indicated a potential hydroxamate-family siderophore. Liquid chromatography (LC)-ES-MS analysis of secondary metabolites secreted by *G. adspersum* into the growth medium under iron-limiting conditions revealed a group of previously undescribed siderophores. Genome and MS/MS analysis suggested that these structures might be related to the coprinoferrin family of siderophores. Aside from siderophores, the genome and LC-MS analysis revealed *G. adspersum* to be a prolific producer of a variety of triterpenoids and sesquiterpenoids, in agreement with previous findings. This is the first description of the genome sequence of *G. adspersum* and its siderophores.

## Introduction

*Ganoderma* is a basidiomycete belonging to the family *Ganodermataceae* (order *Polyporales*). They form perennial, bracket-shaped to crust-like fruiting bodies and cause a white rot in certain broad-leaved trees [1]. Of the more than 300 species of *Ganoderma*, they are considered either saprophytes (fungi that degrade dead wood using lignocellulose enzymes) or hemibiotrophs (plant pathogens) that attack living trees [2,3]. In pharmacological studies, various chemicals have been extracted from *Ganoderma* species including the triterpenoids 12-hydroxyganoderic acid and ganorbiformin and the alkaloid lucidimines [4]. *Ganoderma* is a well-known producer of secondary metabolites including triterpenes, polysaccharides (heteroglycans or beta-d-glucans) and peptidoglycans [4].

One understudied metabolic pathway among the *Ganoderma* and basidiomycetes in general is the siderophore metabolic pathway. Siderophores are iron uptake molecules that scavenge iron when it is limited. Iron is an essential nutrient to nearly all life forms, including fungi. During growth and development of a cell, iron can serve as a co-factor for proteins that are needed for basic metabolic functions like DNA and amino acid synthesis and electron transport. Although iron is abundant in the environment, its bioavailability is low because it is rapidly oxidized from ferrous iron (Fe^2+^) to ferric iron (Fe^3+^) under oxic conditions, which then forms particulate iron oxyhydroxides or binds strongly to organic matter.

Bacteria and fungi can secrete siderophores to scavenge and uptake iron. The siderophore can either be reabsorbed by the same cell that secreted it, or it can be captured by neighbouring micro-organisms including other bacteria and fungi. In some cases, fungi can utilize bacterial siderophores [5]. Some pathogenic fungi and bacteria possess siderophores that bind iron from mammalian host iron storage proteins like ferritin, haem and transferrin [6,7]. But not all fungi are capable of this function. Fungal siderophore structures commonly involve hydroxamate or carboxylate chelating moieties [8].

 *Ganoderma adspersum* (Schulzer) Donk is a white-rot fungus capable of degrading wood using lignocellulose enzymes. It can be distinguished from other *Ganoderma* spp. such as *Ganoderma lipsiense* by the size of the base of their fruiting body, which is thicker (40–100 mm), with a downward attachment to host trees (e.g. decurrent attachment). When viewed under the microscope, the basidiospores are larger (8.5×12×8 µm) compared to *G. lipsiense* (7×9×4.5–6.0 µm) [1].

 Although several *Ganoderma* species have been sequenced, there is no genome description for *G. adspersum*. In this study, we investigated the genome of *G. adspersum* for the presence of genes potentially linked to iron acquisition. Additionally, secondary metabolites from cultures of *G. adspersum* grown under iron-limited conditions were investigated.

## Methods

### Growth conditions and culture identification

*G. adspersum* was collected and isolated from a basidiocarp growing on a beech tree in Freiburg, Switzerland. The culture was deposited in the Collection of Westerdijk Fungal Biodiversity Institute CBS 147723 *G. adspersum*. For genome sequencing, the culture was grown on 2% malt extract liquid media. The culture was shaken at 120 r.p.m. for a few days at ambient temperature before the pellets were harvested for DNA and RNA extraction.

### DNA extraction and sequencing

DNA was extracted by Biolytx AG (Witerswill, Switzerland) using the AllPrep Bacterial/Fungal DNA/RNA/Protein Kit (QIAGEN). DNA was eluted in 50 µl elution buffer according to the manufacturer’s instructions. DNA extracted samples were sent to the Joint Genome Institute (JGI) for draft genome sequencing. Samples were sequenced at JGI using PacBio. An input of 50 ng of genomic DNA was sheared to 6–10 kb using the Megaruptor® 3 (Diagenode). The sheared DNA was treated with exonuclease to remove single-stranded ends, DNA damage repair enzyme mix, end-repair/A-tailing mix and ligated with amplification adapters using SMRTbell Express Template Prep Kit 2.0 (PacBio) and purified with ProNex® Size-Selective Purification System (Promega). The purified ligation product was split into two reactions and enriched using 10–18 cycles of PCR using SMRTbell® gDNA Sample Amplification Kit (PacBio). The amplified product was combined and treated with DNA damage repair enzyme mix, end-repair/A-tailing mix and ligated with barcoded overhang adapters. Up to 16 libraries were pooled in equimolar concentrations, and the pooled libraries were size-selected using the 0.75% agarose gel cassettes with Marker S1 and High Pass protocol on the BluePippin (Sage Science). PacBio Sequencing primer was then annealed to the SMRTbell template library, and sequencing polymerase was bound to it using Sequel II Binding Kit 2.0. The prepared SMRTbell template libraries were then sequenced on a Pacific Biosystems' Sequel IIe sequencer using SMRT Link 10.2, tbd-sample dependent sequencing primer, 8M v1 SMRT cells and version 2.0 sequencing chemistry with 1×1800 sequencing movie run times.

### RNA extraction, sequencing and assembly

RNA was extracted by Biolytx AG (Dittingen, Switzerland) using the AllPrep Bacterial/Fungal DNA/RNA/Protein Kit (QIAGEN). RNA was eluted in 50 µl elution buffer according to the manufacturer’s instructions. RNA extracted samples were sent to the JGI for transcriptome sequencing. Plate-based RNA sample prep was performed on the PerkinElmer Sciclone NGS robotic liquid handling system using Illumina’s TruSeq Stranded mRNA HT sample prep kit utilizing poly-A selection of mRNA following the protocol outlined by Illumina in their user guide, https://support.illumina.com/sequencing/sequencing_kits/truseq-stranded-mrna.html, and with the following conditions, total RNA starting material was 200 ng per sample and 10 cycles of PCR was used for library amplification. The prepared libraries were quantified using KAPA Biosystems’ next-generation sequencing library qPCR kit and run on a Roche LightCycler 480 real-time PCR instrument. Sequencing of the flow cell was performed on the Illumina NovaSeq sequencer using NovaSeq XP V1.5 reagent kits, S4 flow cell, following a 2×151 indexed run recipe. RNASeq reads have been filtered and trimmed for artefact sequence using kmer matching (kmer=25), allowing one mismatch, and the detected artefact was trimmed from the 3′ end of the reads. RNA spike-in reads, PhiX reads and reads containing any Ns were removed. Quality trimming was performed using the phred trimming method set at Q6. Finally, following trimming, reads under the length threshold were removed (minimum length 25 bases or 1/3 of the original read length – whichever is longer). Filtered reads were assembled into a consensus sequence using Trinity v.2.8.5 [9]. Genomic contigs masked using Repeat Masker [10] using Repbase [11] and RepeatScout-derived [12] libraries of fungal repeats. A combination of *ab initio* [FGENESH (Softberry, Inc.) and Genemark [13]], homology-based [FGENESH+(Softberry, Inc.) and Genewise [14]] and transcriptome-based [15] gene predictors was used, and predicted gene models were functionally annotated using SignalP [16], TMHMM [17], InterProScan [18 and blastp [19] against the National Center for Biotechnology Information (NCBI) non-redundant protein dataset, Kyoto Encyclopedia of Genes and Genomes (KEGG) [20] and Eukaryotic Orthologous Groups (KOG) [21].

### Genome assembly and annotation

The artefacts- and mitochondria-filtered CCS reads were assembled with Flye [22] version 2.9-b1768 (-g 100 k --asm-coverage 100 --pacbio-hifi) and polished with two rounds of RACON [23] version 1.4.13 [-u -t 36]. The genome assembly was annotated using the JGI Annotation Pipeline Genome, which utilizes the assembled transcriptome and combines several gene prediction and annotation methods [24,25]. The assembly and annotations are made available in JGI MycoCosm [24] (https://MycoCosm.jgi.doe.gov/Ganads1) as well as deposited to NCBI (Bioproject PRJNA1080951 and Biosample SAMN40290235). The MycoCosm online fungal database was used to analyse the gene annotation results [26]. Secondary metabolites, based on the genome, were predicted with SMURF [27] as part of the MycoCosm analysis. The program Benchmarking Universal Single-Copy Orthologs (BUSCO v. 5.8.2) [28] was used to assess the quality of the predicted proteome of *G. adspserum* using the `basidiomycota_odb10` lineage dataset in proteome mode.

### Secondary metabolite analysis using antiSMASH

To enhance the detection and analysis of genes potentially involved in secondary metabolite production, antiSMASH, an online tool that predicts secondary metabolites [29] was also used. This tool is specifically designed to identify and analyse biosynthetic gene clusters (BGCs), providing insights into the genomic potential for producing secondary metabolites. Once BGCs are identified, one can further analyse the genes within the cluster using the built-in blastp search. The unmasked assembled genome was submitted to antiSMASH, and it annotated 60 scaffolds within the genome.

### Phylogenetic analysis

Representative amino-acid sequences from the major facilitator superfamily (MFS) were first retrieved from the UniProt database [30]. Only fungal-based sequences with the highest level of experimental evidence were retained. The yeast siderophore-iron transporter ARN1 (UniProt no. P38731 • ARN1_YEAST) was selected as the MFS representative. From the ARN1 entry, an external link in the phylogenomic databases section led to the Orthologous Matrix (OMA) resource, which identifies orthologs across complete genomes [31]. Orthologs of ARN1 were downloaded from OMA group 118032 (fingerprint VPTWPFI, 25 members). For Cpf1 and Cpf2, each protein sequence was retrieved from UniProt (UniProt no A8NF97•CPF1_COPC7 and A8NF99 •CPF2_COPC7) and its orthologous groups located in OMA. Orthologs of Cpf1 came from group 1073105 (fingerprint WQYVPVR, 87 members), and those of Cpf2 came from group 1019604 (fingerprint SFAMYLK, 46 members). Because OMA contained relatively few fungal non-ribosomal peptide synthetases (NRPSs) and terpene synthases, the OrthoDB database (v.12) was used instead [32]. We downloaded the polyporales NRPS orthologous set (14670at5303) and the polyporales terpene orthologous set (54933at5303). All orthologous sequences were aligned, and phylogenetic trees were inferred with the NGPhylogeny.fr ‘one-click’ workflow, which uses PhyML for tree construction by default [33,34].

### Metabolite analysis

For the iron limitation studies, *G. adspersum* was grown on low-nutrient agar (LNA) Petri dishes [35] containing the CAS reagent (chrome azurol) for siderophore tests [36] based on a previously published split Petri dish method [37]. Briefly, 500 ml of LNA media with no Fe contained 2.5 g alpha-d-glucose, 0.0065 g l-asparagine, 0.5 g KH_2_PO_4_, 0.15 g MgSO_4_, 0.25 g KCl, 0.004 g Mn(CH_3_COO)_2_• 4H_2_O, 0.001 g Zn(NO_3_)_2_•6H_2_O, 0.025 g Ca(NO_3_)•4H_2_O, 0.004 g NH_4_NO_3_, 0.001 g CuSO_4_ and 7.4 g agar. This LNA media with no Fe was mixed with 50 ml of a 10X CAS reagent solution after separately sterilizing both reagents (121 °C, at least 40 min) and cooling them to 50 °C. The CAS reagent solution consisted of 25 ml of CAS solution (1.05 mM), 20 ml of hexadecyltrimethylammonium bromide (5 mM) and 5 ml of FeCl_3_ (2.6 mM). Once the media was cooled to 50 °C, it was mixed with 50 ml of CAS solution and then poured into a Petri dish and allowed to solidify. Once solidified, half of the solidified agar was cut out, and LNA agar media (with 0.005 g FeSO_4_/500 ml) was poured into the empty side of the Petri dish. White-rot fungi were inoculated on the side of the Petri dish without CAS (Fig. S1, available in the online Supplementary Material). This method tests the ability of microbes, including filamentous fungi, that are grown under iron-limited conditions, to secrete siderophores into the half of the Petri dish containing CAS [37]. Biomass was scraped off CAS assay plates (two replicate Petri dishes with biomass and a replicate control sample with no fungi) (Fig. S1), and the agar below the biomass was cut out. The agar sample was then extracted by adding 25 ml methanol containing 0.1% formic acid. A control sample, with no fungus, received the same treatment as agar from the biomass above. The mixture was shaken overnight at 120 r.p.m. at room temperature. The next day, the liquid was collected, and the solvent evaporated to obtain dry material for shipment.

### Liquid chromatography-ES-MS

Samples were reconstituted in 0.5 ml of methanol and 3.5 ml of high-purity water (18 MΩ cm). The reconstituted samples were acidified with 0.1% formic acid. Prior to injection, samples were syringe filtered (0.2 µm, PES membrane). Metabolites in the agar extracts were analysed using high-resolution liquid chromatography (LC)-MS/MS (Orbitrap Exploris 480, Thermo Fisher). Sample volumes of 25 µl were injected and separated using a Restek Raptor C18 (2.1×100) column with a flow rate of 0.4 ml min^−1^, and the column compartment was kept at 45 °C. A gradient elution was used with solvents A and B (A: water, 0.1% formic acid, 1% acetonitrile; B: acetonitrile, 0.1% formic acid, 2% water; gradient: 0–1.5 min 0% B, 1.5–8 min 0–100% B; 8–10 min 100% B; re-equilibration at 100% A for 4 min). Samples were desalted on the column by eluting to waste for the first 1.5 min. Samples were injected twice to acquire data in positive and negative ionization modes. Full-scan mass spectra (*m/z*=80–1500) were acquired with the resolution set to *R*=120,000 (full width at half maximum at *m/z*=400) and data-dependent MS/MS acquisition of the top three most abundant ions in each cycle. Product ions were generated in HCD mode with 35 eV collision energy and an isolation window of 1.5 Da. The resolving power for MS/MS analysis was *R*=15,000 (full width at half maximum at *m/z*=400). During the analysis, a prominent ion with *m/z*=202.080 was observed in all MS/MS spectra, independent of the specific precursor ion. This apparent contaminant ion was removed computationally from all MS/MS spectra before further analysis.

Siderophore analysis LC-MS followed previously reported methods [38,39] utilizing (1) the specific ^54^Fe-^56^Fe natural isotope pattern to search for siderophore-Fe complexes, (2) the exact mass difference between the apo siderophore and Fe-complexes and (3) siderophore-specific MS/MS patterns. To analyse non-siderophore exometabolites, all MS1 features (defined by a specific retention time, *m/z* and associated peak areas) with associated MS/MS spectra were extracted using El-Maven for both positive and negative ionization modes [40]. Significant features present in the control agar extract were removed (peak area of feature in control extract >0.01% of feature in sample). The remaining features were used to generate feature-based MS/MS molecular networks in MATLAB with a method described previously [41,42] and visualized in CytoScape [43]. Before the network was calculated, each MS/MS spectrum was filtered to keep only the top five most abundant ions within a 50 Da window and remove ions with less than 0.1% of intensity relative to the most abundant fragment ion. Features represented nodes in the network and were connected by edges if they had a minimum cosine score of *R*≥0.7 and a minimum of 5 matched fragment ions while connecting a maximum of 10 neighbour nodes for each node. Fragment ions of two different precursor ions were matched if they had the same *m/z* value or if the difference between the *m/z* of two fragment ions was the same as the *m/z* difference between their precursor ions. The tolerance was *Δm/z=*5 p.p.m. or 0.003 Da. The Global Natural Products Social Molecular Networking (GNPS) MS/MS database was searched to find potential compound matches [44]. The software tools SIRIUS [42] and Canopus [45] were used in addition to calculate molecular sum formulas, compound classes and to obtain structure suggestions for compounds without GNPS MS/MS matches.

## Results and discussion

### Genome assembly overview

The genome size of *G. adspersum* resulted in 93 Mbp with a sequencing coverage of 88X (Table 1). Previous sequencing efforts of other *Ganoderma* species such as *Ganoderma leucocontextum* [46], *Ganoderma lingzhi* [46] and *Ganoderma australe* [47] were less than 93 Mbp (Table 1). In total, there were 6,680 genes predicted with functional annotation using Gene Ontology [48], 5792 well-characterized genes using KOG annotation and 3,881 functional KEGG annotations, using the filtered models2 option in the Mycososm online portal [49]. In addition to the gene annotations above, 753 genes were annotated as being related to the carbohydrate-active enzymes [50], and included glycoside hydrolase genes, glycosyl transferase genes, polysaccharide lyase genes and carbohydrate esterase genes. Additional gene annotations included 432 peptidase genes, 967 transporter genes and 859 transcription factor genes.

**Table 1.** Genome assembly information

Genome feature	*G. adspersum*	*G. australe canu* ^†*^	*G. leucocontextum* ^*‡^	*G. lingzhi* ^*^
Genome assembly size (Mbp)	92	84.27	60.34	60.56
Sequencing read coverage depth	88×	100×	18.27×	na
Number of scaffolds	586	776	843	342
Scaffold N50	72	17	66	37
Scaffold L50 (Mbp)	0.33	0.2	0.2	0.4
GC (%)	70.39	55.48	55.95	55.88
Largest scaffold(s) (Mbps)	1.48, 1.47, 1.36	1.6	1.72, 1.19, 1.10	2.1
BUSCO proteome (Basidiomycota_odb10)	C: 98.4% D: 8.8% S: 89.6% F: 0.3% M: 1.2%	*C: 84.5% *D: 23.4% S: NA F: NA M: NA	*C: 99.8% D: NA S: NA F: NA M: NA	*C: 73.1% D: NA S: NA F: NA M: NA

*Data from [46].

†Data from [47].

‡Data from MycoCosm.

C, genome completeness; D, duplication; F, fragmented; M, missing; NA, information not available in publications; S, single copy.

The quality of the predicted proteome was assessed using BUSCO and the ‘basidiomycete_odb10’ dataset, which has also been used in previous comparisons of *Ganoderma* genomes [46]. The high completeness score of 98.4%, low fragmentation score (0.3%) and missing score (1.2%) for the *G. adspesrum* predicted proteome indicate that the gene annotations are robust and reliable. The low duplication score of 8.8% indicates that there is minimal redundancy in the predicted proteome, supporting the presence of a predominantly monokaryotic genome assembly. This is in sharp contrast to *G. australe*, in another study, which had a high duplication score of 23% and was predicted to be a dikaryotic genome assembly [46].

### Transport-related genes

Based on the genome annotation results, four genes were annotated as siderophore-related genes, three of the genes were annotated as siderophore uptake or transporter protein genes and one was annotated as an MFS general transporter gene. MFS transporters carry substrates up- or down-concentration gradients using energy stored in electrochemical gradients. If genes were ambiguously annotated, such as being labelled as siderophore-related, those sequences were run through the NCBI blast Protein (blastp) search. This helped to match them to known sequences with specific names or functions. A match using blastp search is defined here as having an *e*-value <0.001 [51] and having a percent identity >35% over 80 aa [52]. Two protein sequences (Ganads1_837952 and Ganads1_892859) that were associated with siderophores, when analysed using blastp, matched to a siderophore transporter (MmpL4) and to a siderophore exporter (MmpL4), related to *Ganoderma boninense* (Table S1). Additionally, two other genes (Ganads1_890411 and Ganads1_506532) matched to siderophore transporters related to *G. boninense* (Table S1). MmpL4 is necessary for siderophore export in the bacterium *Mycobacterium tuberculosis* and is suggested to import them as well [53,54]. To further explore these putative siderophore transporters, we used phylogenetic analysis to study the relationship of these putative transporters to orthologs that form a part of the MFS domain-containing proteins. Three of the protein sequences Ganads1_837952, Ganads1_506532 and Gands1_890411 form clades that appear closely related to the *Candida glabrata* NRRL Y65 lineage, while Ganads1_892859 clustered with more distantly related yeast lineages including *Candida albicans* (Fig. S2). Furthermore, we used blastp comparison between siderophore transporter-related genes (with experimental confirmation) and the *Ganoderma* proteome (Table S2). However, the blastp search did not yield any matches to the *Ganoderma* proteome. Based on these combined analyses, we can only speculate that these proteins are involved in transport, but they give no indication of siderophore transport.

### Secondary metabolite predictions

**Fig. 1.**
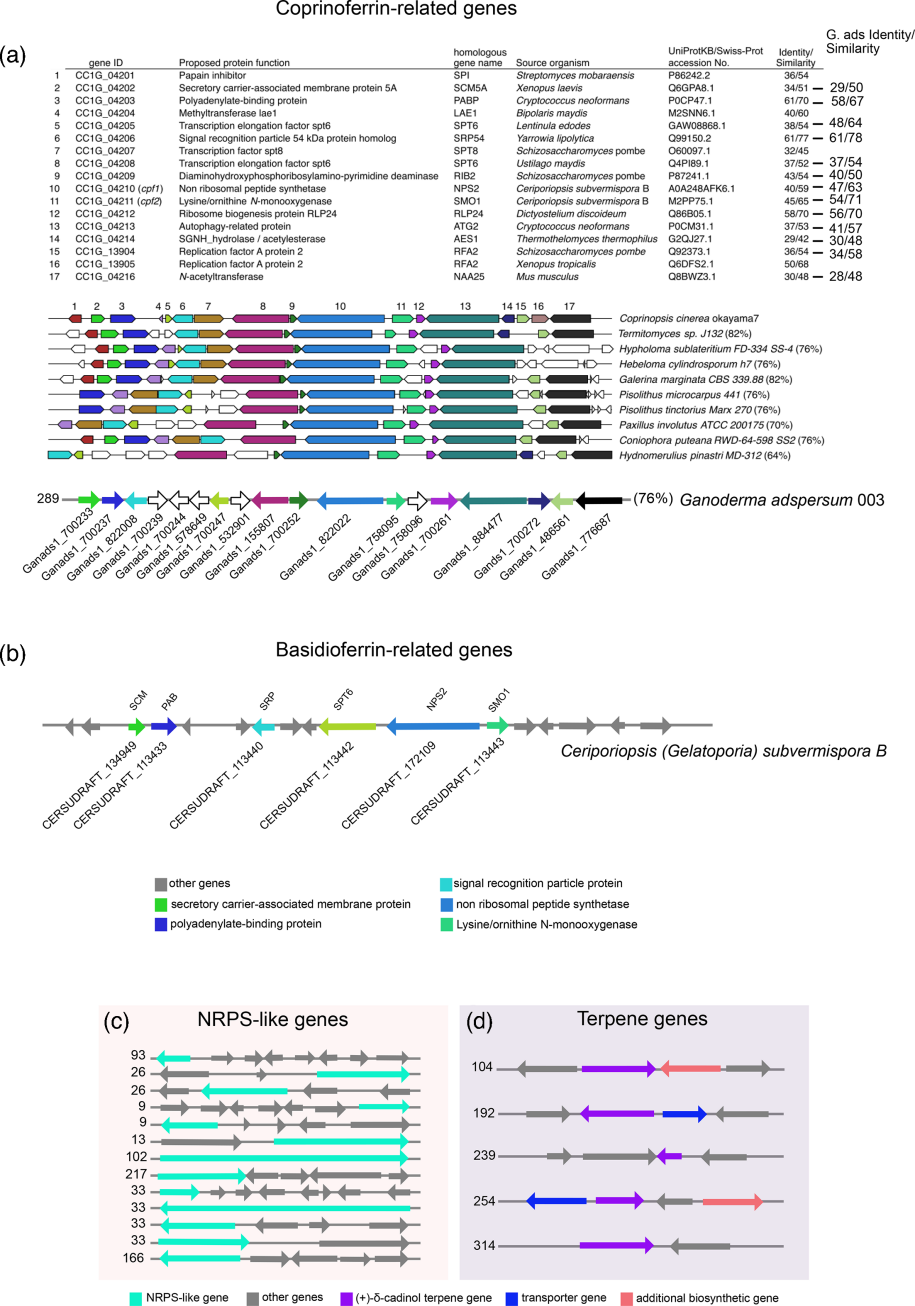
Metabolic gene clusters of *G. adspersum*. (a) Putative coprinoferrin gene cluster based on synteny with clusters identified by Tsunematsu *et al*. The different colours correspond to the different genes (1–17) listed in the table. Percent similarity in the table refers to amino acid sequence identity between the genes in the gene cluster of *Coprinopsis cinerea* and the homologous genes identified in other basidiomycete genomes including *G. adspersum*. Only cpf1 (NRPS) and cpf2 (monooxygenase) are predicted to be directly involved in siderophore biosynthesis. Other genes are included based on conserved genomic context. Functional linkage has not yet been experimentally validated for any of these genes. Seventy-six per cent of the genes in the putative coprinoferrin gene cluster were also present in *G. adspersum*, as indicated. Part of figure A was adapted with permission from Tsunematsu, Yuta, *et al.* “Genomic mushroom hunting decrypts coprinoferrin, a siderophore secondary metabolite vital to fungal cell development”. Organic letters 21.18 (2019): 7582–7586. ©2019 American Chemical Society. (b) Putative basidioferrin gene cluster based on antiSMASH analysis of the *C*. (*Gelatoporia*) *subvermispora* B genome. (c) NRPS-like genes were present on scaffold 93 (Ganads1_864039), scaffold 26 (Ganads1_548478 and Ganads1_548544), scaffold 9 (Ganads1_862379 and Ganads1_740093), scaffold 13 (Ganads1_741038), scaffold 102 (Ganads1_878671), scaffold 217 (Ganads1_756141), scaffold 33 (Ganads1_550438, Ganads1_177632 and Ganads1_828679) and scaffold 166 (Ganads1_754268). (d) Several terpene genes were found on scaffold 104 (Ganads1_517111), scaffold 192 (Ganads1_690129), scaffold 239 (52108–53918, excluding introns), scaffold 254 (Ganads1_817038) and scaffold 314 (Ganads1_827016). This figure is not to scale.

To identify genes potentially involved in siderophore synthesis, we compared the genome of *G. adspersum* to two gene clusters from known siderophore-producing model basidiomycete fungi: one from the coprinoferrin-producing basidiomycete *Coprinopsis cinerea* (Fig. 1a), and the other from the basidioferrin-producing *Ceriporiopsis* (*Gelatoporia*) *subvermispora* B (Fig. 1b) [55,56]. A comparative analysis revealed that 76% of the genes in the putative coprinoferrin biosynthetic cluster were also present in the *G. adspersum* genome (Fig. 1a)*,* while 35% of the genes in the basidioferrin cluster were identified (Fig. 1b).

Coprinoferrin is a recently discovered tripeptide tris-hydroxamate siderophore in which each of the hydroxamate nitrogens is acylated with hexanoyl groups [55,57, 58]. The core structure of coprinoferrin is similar to basidioferrin, which is also a tris-hydroxamate, but the hydroxamate nitrogen groups are acetylated, as shown in Fig. S9 [56]. Both gene clusters for coprinoferrin and basidioferrin have two core genes present that include a type VI NRPS and a lysine/ornithine N-monooxygenase. NRPS enzymes are multifunctional enzymes that assemble the backbone of different secondary metabolites in fungi including siderophores [59,60]. Lysine/ornithine N-monooxygenases are key in the oxygen-dependent hydroxylation of the amino acid groups of lysine or ornithine to form the iron-binding hydroxamic acid groups during siderophore biosynthesis [61]. In the *G.adspersum* genome, antiSMASH identified the NRPS core gene as Ganads1_822022 and the lysine/ornithine N-monooxygenase gene as Ganads_758095 located next to Ganads1_822022 on scaffold 289.1 (Fig. S3A–C).

In the coprinoferrin biosynthesis pathway of the model basidiomycete fungus *Coprinus cinereus*, three main steps are involved: the hydroxylation of ornithine to N(5)-hydroxyornithine, catalysed by the monooxygenase Cpf2 [55], the acylation of N(5)-hydroxy-l-ornithine to N(5)-hexanoyl-N(5)-hydroxyl-l-ornithine, which is catalysed by an unknown acyltransferase [55], and finally the amide bond formation between three N(5)-hexanoyl-N(5)-hydroxyl-l-ornithine molecules catalysed by the NRPS Cpf1, resulting in the linear trimer [55]. Tsunematsu *et al*. [55] genetically, metabolically and functionally characterized Cpf1, primarily through *in silico* and domain-based analyses, and determined based on homology that it was a novel NRPS. Additionally, they inferred, based on homology, that Cpf2 could be a monooxygenase. Thus, these genes, which form a part of a proposed biosynthetic cluster, await experimental validation.

Using the MycoCosm blastp tool (filtered model proteins setting), we compared Ganads1_822022 with Cpf1 (UniProt A8N897). The alignment covered 79% of the query, with 45.4% identity and an *e*-value of 2.09 E−78. Similarly, a comparison of Ganads1_758095 with Cpf2 (UniProt A8NF99) resulted in a query with 62.8% query coverage, 55.8% identity and an *e*-value of 1.27 E−75. Furthermore, analysis of the genome assembly and the gene annotations for neighbouring genes on scaffold 289.1 revealed matches to genes previously identified as part of the coprinoferrin biosynthesis pathway in other fungal genomes [55] (Fig. 1a). This finding suggests that the gene cluster is evolutionarily conserved. Interestingly, homologous NRPS–ornithine hydroxylase gene pairs, conserved across various basidiomycetes, are classified as a common basidiomycete siderophore BGC [55]. Additionally, the cluster includes genes encoding transcription factors, replication factors and autophagy-related proteins, which may suggest roles in developmental and cellular processes beyond siderophore biosynthesis (Fig. 1b) [55]. However, certain genes in the *G. adspersum* cluster did not match any previously described genes within this pathway. These differences may account for structural variations in the coprinoferrin siderophore synthesized by *G. adspersum*, suggesting that it may not exactly match the structures described in earlier studies.

Initial blastp analysis within antiSMASH showed that Ganads1_822022 was similar to a type VI NRPS from *C*. (*Gelatoporia*) *subvermispora* featuring three predicted amino acid domain sequences (Fig. S3B). Each domain sequence was analysed individually using the blastp search function within antiSMASH. The first domain matched a specific part of a type VI NRPS sequence (Table S2). This alignment corresponded to the CsNPS2 gene from the basidiomycete fungus *C*. (*Gelatoporia) subvermispora* (Fig. 1b) [56]. CsNPS2 (UniProt A0A248AFK6) is an NRPS that has been experimentally verified. blastp searches on the second and third domain amino acid sequences matched to different parts of CsNPS2 (Table S2). All three domains fall within carrier protein regions within CsNPS2, as indicated by UniProt domain annotations. Furthermore, when CsNPS2 was initially characterized, it was found to aid in the synthesis of a trimeric-hydroxamate (or tris-hydroxamate) siderophore [56].

To explore if other genes might play a role in this biosynthetic pathway, we also examined the original genome context in which CsNPS2 was described using antiSMASH (*C. subvermispora* genome, GenBank ID AEOY00000000.1). This analysis revealed that the corresponding gene cluster includes a lysine/ornithine N-monooxygenase. However, no acyltransferase or acetyltransferase gene was identified within the cluster (Fig. 1b) [56]. This suggests that although the genes required for hydroxamate formation are co-localized, the enzyme responsible for *N*-acetylation of the monomers may be encoded elsewhere in the genome or remains to be identified.

### Comparison to experimentally verified siderophores

To further investigate the functional identity of the putative monooxygenase gene Ganads1_758095, we conducted additional blastp searches against experimentally validated siderophore biosynthesis enzymes from model fungal species (Table S1). This analysis revealed that Ganads1_758095 shares 48.9% identity with Sid1 from *Mycosarcoma maydis* and 59% identity with SidA from *Aspergillus fumigatus* [62,63]. Both enzymes are well-characterized l-ornithine N^5^-monooxygenases involved in hydroxamate siderophore biosynthesis. Sid1 catalyses the conversion of l-ornithine to N^5^-hydroxyornithine, the first key step in hydroxamate formation. Similarly, SidA is a l-ornithine N^5^-monooxygenase that performs the same oxygenation step and is essential for siderophore production in *A. fumigatus*. This sequence similarity between Ganads1_758095 and these experimentally verified monooxygenases further supports the hypothesis that Ganads1_758095 encodes a functionally conserved l-ornithine N_5_-monooxygenase in *G. adspersum*.

### Phylogenetic comparisons

To study the evolutionary relationships of these NRPS genes to other basidiomycetes, we performed phylogenetic analysis of Ganads1_822022 and Ganads1_758095 by clustering each amino acid sequence with orthologous groups containing representative sequences homologous to Cpf1 or Cpf2. We found that Ganads1_822022 clustered with NRPS sequences from other basidiomycetes fungi, specifically grouping with *Dichomitus squalens* strain LYAD 421 and *Grifola frondosa* NRPS2. It was more distantly related to a neighbouring subcluster that includes *Gelatoporia subvermispora* B and Cpf1 (*Coprinopsis cinerea*, sequence ID COPC708040), along with other Agaricomycete NRPS sequences (Fig. S4). Ganads1_758095 clustered within a basidiomycete group that also included Cpf2 (*Coprinopsis cinerea*, COPC102790), as well as monooxygenase-like sequences from *D. squalens*, *Grifola frondosa* and *Trametes* species (Fig. S5). This close phylogenetic relationship suggests that Ganads1_758095 may share a conserved evolutionary origin with these fungal monooxygenases, including Cpf2. Currently, coprinoferrin or structural analogues have not been isolated from *G. adspersum* or other fungi besides *Coprinospsis cinerea*.

Other NRPS-like genes present in the annotated genome are shown in Fig. 1c. We also performed a phylogenetic analysis of these NRPS-like protein sequences by comparing them to NRPS orthologues from other fungal genomes. The resulting tree showed that the *G. adspersum* NRPS-like sequences formed multiple subclusters (Fig. S6). One subcluster containing Ganads1_754268, Ganads1_864039 and Ganads1_548544 grouped more closely with NRPS genes from *Grifola frondosa* and *Gelatopora suvermispora* B while still forming a separate group. This suggests that while these protein sequences may share an evolutionary origin, they could differ in function. In contrast, the remaining *G. adspersum* NRPS sequences were more closely related to each other than to any of the reference fungal NRPS sequences, suggesting that they have evolved uniquely within *G. adspersum*, separate from other fungi.

Additionally, antiSMASH predicted 27 BGCs annotated as terpenes. Terpenes are organic compounds composed of isoprene units that are produced by plants, animals and fungi. Of the 27 predicted BGCs, 5 contained clusters with matches to (+)-δ-cadinol terpenes (Fig. 1d). Fungal terpenes could be involved in chemical defence, communication and signalling, adaptation to the environment or act as aromatic compounds [64]. Cadinol terpenes are used as anti-breast cancer and anti-inflammatory compounds [65]. blastp analysis of the five genes resulted in several genes with matches to terpene synthases, enzymes which help synthesize terpenes from isoprenoids [4] (Fig. 1d, Table S2). Phylogenetic analysis of these terpene synthase sequences with terpene synthase orthologs from other fungal genomes revealed that the terpene synthases formed two separate clusters. One cluster, containing Ganads1_690129 and Ganads1_695714, grouped more closely with terpene synthases from *Ganoderma sinense*, *G. leucocontextum* and *D. squalens*, suggesting these terpene synthases may be more conserved among these fungi (Fig. S7). Another cluster containing Ganads1_517111, Ganads1_817038 and Ganads1_827016 formed a separate group, suggesting possible evolutionary divergence of these terpene synthases or perhaps representing a novel group of terpenes within *G. adspersum*.

### Siderophore analysis by LC-MS

**Fig. 2.**
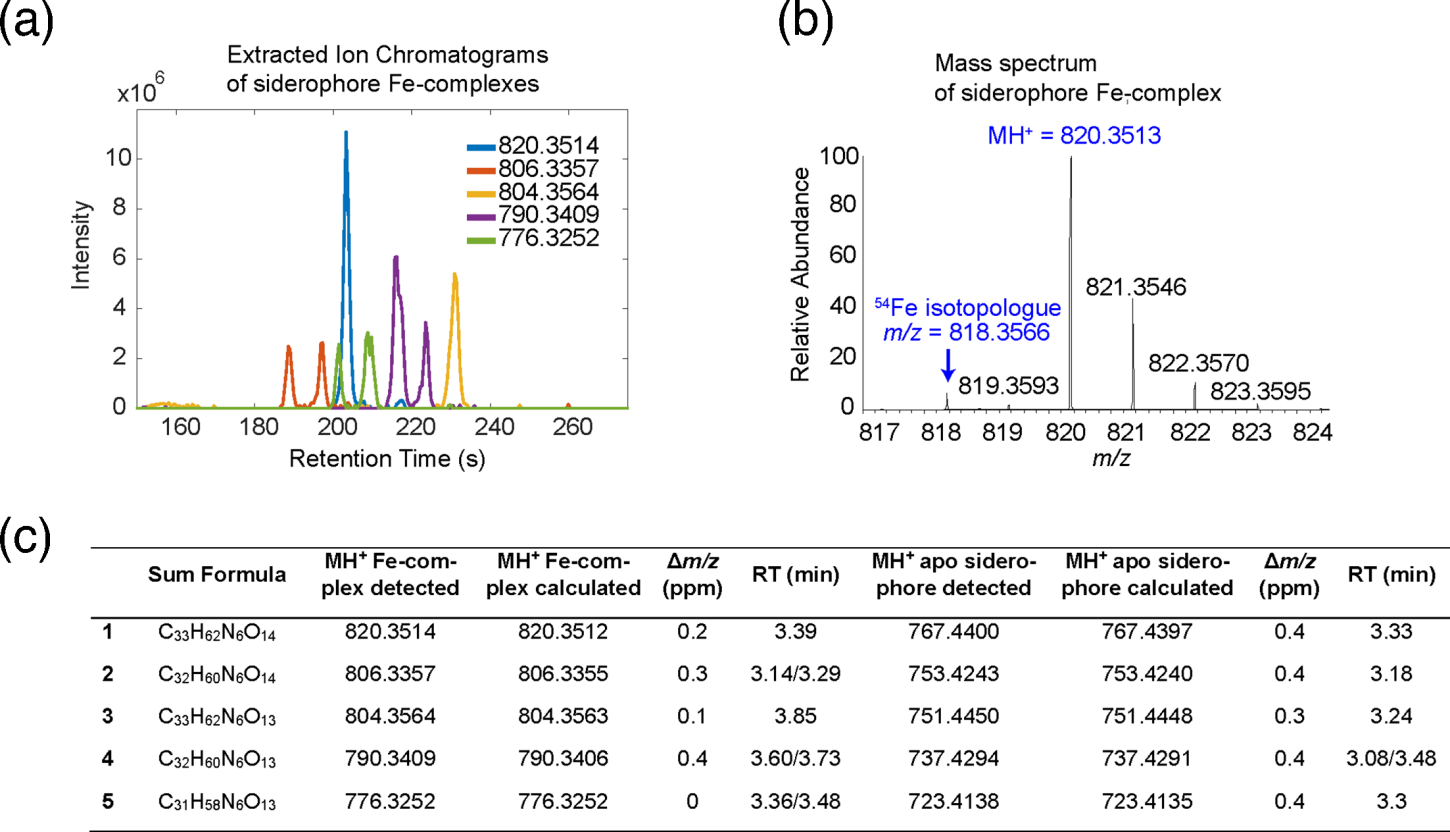
(a) Detected iron-siderophore complexes in incubated iron-limited agar media extracts. (b) An exemplary iron isotope pattern associated with each of the detected siderophores. (c) Elemental sum formulas for the neutral siderophore (M), along with measured and calculated *m/z* values for the MH+ species of the siderophore iron complexes and the associated apo siderophores.

Analysis of agar extracts of *G. adspersum*, grown on iron-limited medium, revealed the presence of five major siderophores with unique masses and retention times (Fig. 2a). An iron isotope pattern was associated with each of the detected siderophores (Fig. 2b). The features were characterized by the presence of both the apo-siderophore and corresponding Fe complexes (Fig. 2c), with the major fraction present as the Fe complex and a minor fraction detected in the apo form. The iron complexes had shifted retention times compared to the apo siderophore species, indicating that Fe complexes remained intact during chromatography with a 0.1% formic acid buffer. The acid stability of the complexes during LC-MS was in agreement with the expected behaviour of tris-hydroxamate siderophores [39]. The calculated elemental sum formulas suggested a family of structures that differed in their formula by -CH_2_- (e.g. methylation, elongated alkyl chain: siderophores 1/2; 3/4; 4/5, Fig. 2c) and -O- (e.g. hydroxylation: siderophores 1/3; 2/4, Fig. 2c). To our knowledge, none of the detected precursor masses corresponded to any known siderophores. An MS/MS spectrum was collected of the most abundant of the five apo siderophores (MH^+^=751.4450). Fragment ions (Table S3, Fig. S8) and neutral losses (Table S4, Fig. S8) were compared to possible fragments of known peptidic siderophores. The fragmentation was in agreement with hydroxamate siderophores containing N^5^-hydroxyornithine residues (fragment ion *m/z*=131.0816, neutral loss *m/z*=130.0731, N^5^-formyl-N^5^-hydroxy-ornithine fragment ion *m/z*=157.0609 and neutral loss *m/z*=158.0686, Tables S3 and S4). The bioinformatic analysis indicated the siderophore was likely in the ferrichrome family and similar to basidioferrin or coprinoferrin (Fig. S9).

The siderophore in this study had prominent fragments and neutral losses in agreement with the sum formula C6H10O2 (fragment ion *m/z* = 115.0759, neutral loss *m/z* = 114.0681). This fragment was not consistent with the structure of basidioferrin (Fig. S9A) which does not contain fragments or neutral losses that have sum formulas involving six carbon atoms without containing nitrogen. The coprinoferrin structure (Fig. S9B) includes terminal N-hexanoyl residues. The mentioned C6H10O2 fragments and neutral losses were consistent with hydroxylated terminal N-hexanoyl residues in a possible structure similar to coprinoferrin. This was also supported by the presence of genes for an acyl transferase, which is hypothesized to be essential for the biosynthesis of coprinoferrin and found in the coprinoferrin gene cluster but not in that of basidioferrin (Fig. 1a) [55]. Closer inspection of the MS/MS spectra further showed that the observed most prominent fragments and neutral losses had sum formulas containing C11, C6 and C5, which were in agreement with the coprinoferrin topology (Figs S8 and S9B).

Coprinoferrin has the elemental sum formula C_35_H_65_N_7_O_10_ and the closest sum formula of the siderophores in this study was C_33_H_62_N_6_O_13_. Therefore, further structural modifications beyond simple hydroxylation of the coprinoferrin structure were required if the novel siderophore in this study was indeed a coprinoferrin analogue. The most abundant and smallest fragment in the MS/MS spectrum (*m/z*=86.0601) had the sum formula C_4_H_7_NO, and this fragment could not be explained by hydroxylation or similar simple modifications of the coprinoferrin structure. The differences between the new siderophore in this study and coprinoferrin could not be fully resolved based on the MS/MS fragmentation. Taken together, the siderophores detected in * G. adspersum* appear to be chemically distinct from basidioferrin and show greater structural similarity to coprinoferrin, albeit with additional modifications. Thus, the available genomic and MS/MS results suggest that *G. adspersum* produces a previously undescribed siderophore structure that may have similarity to the coprinoferrin family of siderophores.

### Analysis of other secondary metabolites by LC-MS

**Fig. 3.**
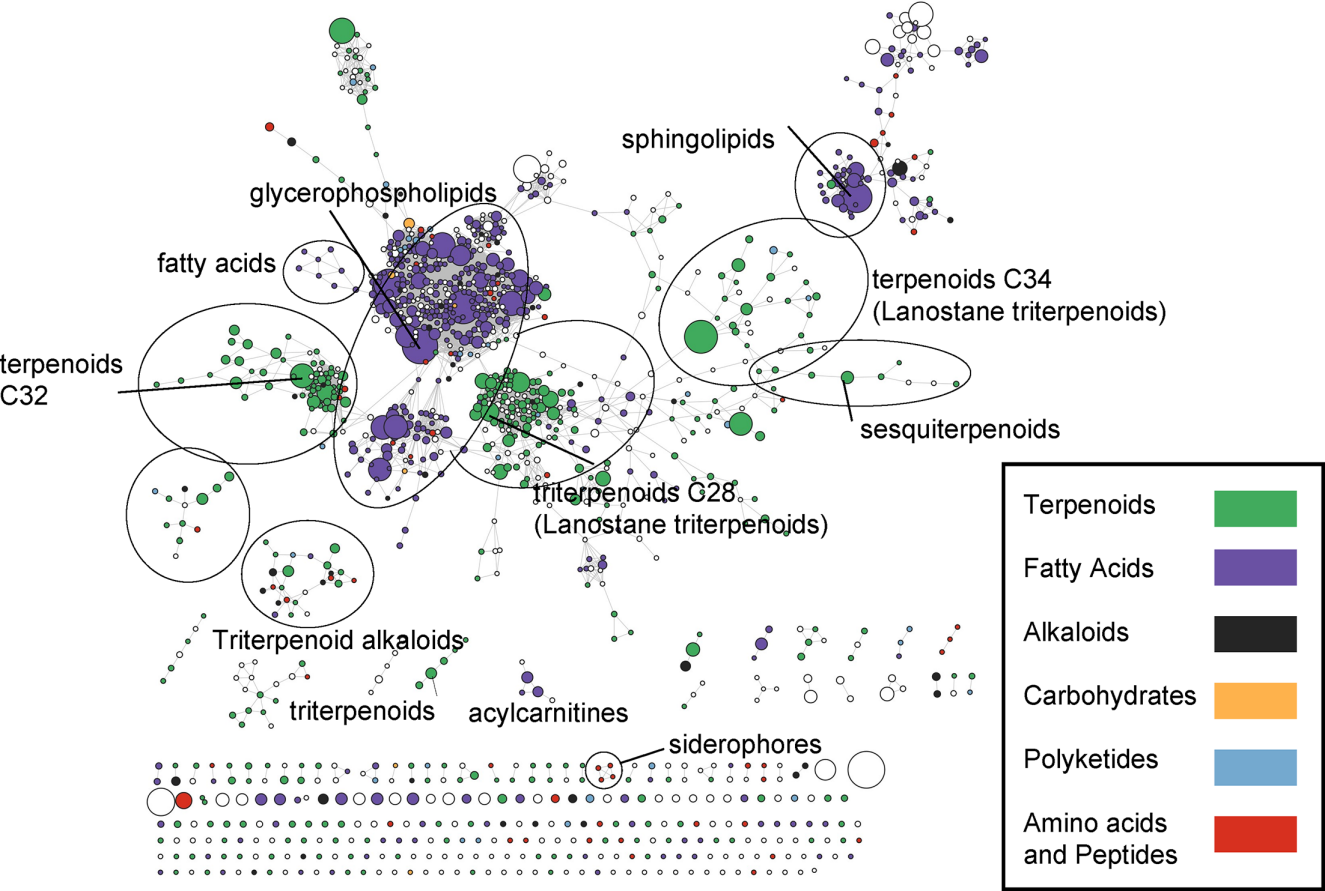
Feature-based MS/MS molecular network, illustrating clusters of structurally related extracellular *G. adspersum* metabolites. The size of nodes illustrates feature peak areas. A supplementary Excel table lists all detected features. For details about the method and parameters used for creating the network, see the ‘Methods’ section.

Alongside siderophores, the LC-MS/MS data showed several other secondary metabolites. An untargeted analysis was performed focusing on all MS1 features with associated MS/MS spectra. Feature-based molecular networking revealed clusters of compounds with similar MS/MS fragmentation patterns (Fig. 3). Compound classes were annotated with the software tool SIRIUS and a GNPS MS/MS library search was done to find known metabolites matching to GNPS library spectra. A list of all detected features and annotations is available as a supplemental table. The detected compound classes consisted primarily of a variety of terpenoids and lipids. Ganoderma fungi are known to produce diverse triterpenoids, including lanostane terpenoids and others, that have previously been classified into structures containing C31, C30, C29, C27, C25 or C24 skeleton carbons [4]. The triterpenoid clusters in this study were likely lanostane triterpenoids based on SIRIUS *in silico* fragmentation predictions. A GNPS library search also suggested the presence of analogues of fusidic acid. Aside from triterpenoids, antiSMASH analysis showed clusters with matches to (+)-δ-cadinol terpenes which are sesquiterpenoids. The LC-MS analysis showed a cluster of sesquiterpenoids including a compound with the best match to deoxyuvidin B (*m/z*=253.1799, RT=3.03 min), a sesquiterpenoid with structural similarity to cadinol and known to be produced by fungi. Several other sesquiterpenoids in this MS/MS cluster were structurally related to this metabolite and differed in their elemental formula by CH_2_, CH_4_O and H_2_. Among the lipids were GNPS library matches to glycerophosphoethanolamine as well as analogues of a variety of phosphatidylcholines and sphingolipids. Additionally, several acylcarnitines differed in their acyl groups. Most of these lipids are common in fungi.

## Conclusions

The genome of *G. adspersum* was sequenced, leading to the identification of a novel siderophore family. While the structure appears to share similarities with coprinoferrin, further studies will be needed to isolate and characterize the compound. The detection of siderophores and the presence of a conserved BGC highlight the potential significance of iron acquisition in the physiology and ecological adaptation of *G. adspersum*. These findings underscore the need for future research to elucidate the functional roles of iron metabolism in this biotechnologically and ecologically important fungus.

## Supplementary material

10.1099/mic.0.001621 Uncited Supplementary Material 1.

10.1099/mic.0.001621 Uncited Table S1.

## Abbreviations

BGCs: biosynthetic gene clusters
BUSCO: Benchmarking Universal Single-Copy Orthologs
GNPS: Global Natural Products Social Molecular Networking
JGI: Joint Genome Institute
KEGG: Kyoto Encyclopedia of Genes and Genomes
KOG: Eukaryotic Orthologous Groups
LC: liquid chromatography
LNA: low-nutrient agar
MFS: major facilitator superfamily
NCBI: National Center for Biotechnology Information
NRPSs: non-ribosomal peptide synthetases
OMA: Orthologous Matrix
