# Supplementary material for: Siderophores and secondary metabolites produced by Ganoderma adspersum
Source: Microbiology (Reading). 2025 Nov 14;171(11):001621. doi: 10.1099/mic.0.001621 (PMC12617850; doi:10.1099/mic.0.001621)
Supplement: Uncited Supplementary Material 1. [file mic-171-01621-s001.pdf]

Siderophores and secondary metabolites produced by *Ganoderma adspersum*

[1]Carolina Reyes, [4]Steven Ahrendt, [4]Robert Riley, [4]Anna Lipzen, [4]Vivian Ng, [4,5]Igor V. Grigoriev, [2]Francis W.M.R. Schwarze, [3]Oliver Baars

[1]Laboratory for Cellulose and Wood Materials, Empa, Überlandstrasse 129, 8600 Dübendorf, Switzerland

[2]Laboratory for Cellulose and Wood Materials, Empa, Lerchenfeldstrasse 5, 9014 St. Gallen, Switzerland

[3]North Carolina State University, Department of Entomology and Plant Pathology, 840 Main Campus Drive, Raleigh, NC, 27695, USA

[4] U.S. Department of Energy Joint Genome Institute, Lawrence Berkeley National Laboratory, Berkeley, CA 94720, USA

[5] Department of Plant and Microbial Biology, University of California, Berkely, CA 94720, UDA

Co-corresponding authors:(CR) carolina.reyes@empa.ch, (OB) olibaars@gmail.com

Table S1. BLASTp comparison of experimentally verified proteins involved in siderophore uptake and biosynthesis. Sequences for comparison were obtained through the UniProt online portal having an annotation score of 3 or higher.

**Sequence used in BLASTp comparison to *G. adspersum* proteome**	**Comments**	**Gads proteome matches**
Mycosarcoma maydis<br>Sid1<br>P56584_SIDA_MYCMD L-ornithine N(5)-monooxygenase	Catalyzes the conversion of L-ornithine to N_5-hydroxyornithine, the first step in the biosynthesis of all hydroxamate-containing siderophores, such as ferrichrome.	Gads758095<br><br>48.9% Identity
Aspergillus fumigatus<br>SidA<br>E9QYP0_SIDA_ASPFU L-ornithine N(5)-monooxygenase	L-ornithine N_5-monooxygenase; part of the siderophore biosynthetic pathway (PubMed:15504822, PubMed:16113265, PubMed:17845073, PubMed:20614882, PubMed:20650894, PubMed:22465572).	Gads758095<br><br>59% identity
Aspergillus fumigatus<br>SidF<br>Q4WF55_SIDF_ASPFU N(5)-hydroxyornithine:cis-anhydromevalonyl coenzyme A-N(5)-transacylase	Hydroxyornithine transacylase; part of the siderophore biosynthetic pathway (PubMed:17845073, PubMed:23617799).	No matches
Aspergillus fumigatus<br>SidH<br>Q4WF54_SIDH_ASPFU Mevalonyl-coenzyme A hydratase	Mevalonyl-coenzyme A hydratase; part of the siderophore biosynthetic pathway (PubMed:22106303).	No matches
Saccharomyces cerevisiae<br>Arn1<br>P38731_ARN1_YEAST Siderophore iron transporter	Involved in the transport of siderophore ferrichrome and so has a role in iron homeostasis.	No matches
Schizosaccharomyces pombe<br>Str3<br>Q92341_STR3_SCHPO Low affinity heme transporter str3	Low affinity heme transporter involved in the assimilation of exogenous heme during conditions of low cellular iron.	No matches
Saccharomyces cerevisiae<br>ENB1<br>Q08299_ENB1_YEAST Siderophore iron transporter ENB1	Involved in the transport of siderophore enterobactin and so has a role in iron homeostasis.	No matches

Aspergillus fumigatus<br>MirB<br>Q4WF31_MIRB_ASPFU MFS siderochrome iron transporter B	Major facilitator transporter involved in triacetylfusarinine C (TAFC) uptake (PubMed:22903978, PubMed:36125294).	No matches
Schizosaccharomyces pombe<br>Str1<br>O74395_STR1_SCHPO Siderophore iron transporter 1	Involved in the transport of siderophore iron and so has a role in iron homeostasis	No matches
Aspergillus fumigatus<br>Sit1<br>Q4WGS5_SIT1_ASPFU Siderophore iron transporter 1	Major facilitator transporter involved in ferrichrome (FC) and ferrioxamine B (FOB) uptake (PubMed:26929401).	No matches
Aspergillus fumigatus<br>Sit2<br>Q4WGE2_SIT2_ASPFU Siderochrome iron transporter 2	Major facilitator transporter involved in ferrichrome (FC) uptake (PubMed:26929401).	No matches

Table S2. ***G.adspesrum*** **sequences and different steps used in verifying their identity.**

Gene ID	Myco. Annotation	Myco. Top Hit (ID)	Myco. Hit Organism	Alt. tool Top Hit (ID)	Alt. Tool Hit Organism	Annotation Approach	Related Figure(s)
Gands1_837952	Siderophore exporter MmpL4	VWO99247.1 MmpL4 (48%)	*Ganoderma boni-nense*	MFS domain contain-ing protein	*NA*	Mycocosm + BLASTp + phylogenetics	Figure S3
Gands1_892859	Siderophore exporter MmpL4	VWO99247.1 MmpL4 (49%)	*Ganoderma boni-nense*	MFS domain contain-ing protein	*NA*	Mycocosm + BLASTp + phylogenetics	Figure S3
Gands1_890411	Siderophore transporter	VWP00770.1 Sidero-phore transporter (81%)	*Ganoderma boni-nense*	MFS domain contain-ing protein	*NA*	Mycocosm + BLASTp + phylogenetics	Figure S3
Gands1_ 506532	Hypothetical protein	VWO99247.1 MmpL4 (71%)	*Ganoderma boni-nense*	MFS domain contain-ing protein	*NA*	Mycocosm + BLASTp + phylogenetics	Figure S3
*Gands1_700233	SCAMP family-domain containing protein	PIL24020.1 hypothetical protein GSI_13771 (96%)	*Ganoderma sinense ZZ0214-1*	KAI1795853.1 scamp-domain-con-taining protein (95%)	*Ganoderma leucocontex-tum*	Mycocosm + BLASTp	Figure 1A
*Gands1_700237	hypothetical protein	VW098649.1 Uncharac-terized protein (89%)	*Ganoderma boni-nense*	KAI795854.1 Polyad-enylate binding pro-tein (89%)	*Ganoderma leucocontex-tum*	Mycocosm + BLASTp	Figure 1A

*Gands1_700247	domain-containing protein	VW098654.1 WD_REPEATS_REGION domain-containing protein (81%)	*Ganoderma boninense*	XP_027612865.1 Transcription elongation factor SPT6 (50%)	*Sparassis crispa*	Mycocosm + BLASTp	Figure 1A
*Gands1_822008	transporter	PIL24018.1 transporter (97%)	*Ganoderma sinense ZZ0214-1*	KAI1795855.1 signal recognition particle SRP54 subunit, M-domain (97%)	*Ganoderma leucocontextum*	Mycocosm + BLASTp	Figure 2A
*Gands1_155807	SH2 domain domain containing protein	PIL24013.1 hypothetical protein GSI_13764 (95%)	*Ganoderma sinense ZZ0214-1*	KAI1795861.1 transcription elognation factor SPT6 (93%)	*Ganoderma leucocontextum*	Mycocosm + BLASTp	Figure 2A
*Gands1_700252	diaminohydroxyphosphoribosylamino-pyrimidine deaminase	TBU35960.1 diaminohydroxyphosphoribosylamino-pyrimidine deaminase (86%)	*Dichomitus squalens*	KAI1795862.1 diaminohydroxyphosphoribosylamino-pyrimidine deaminase (86%)	*Ganoderma leucocontextum*	Mycocosm + BLASTp	Figure 2A
*Ganads_822022	Non-ribosomal peptide synthetase	PIL24012.1 non-ribosomal peptide synthetase (85%)	*Ganoderma sinesnse ZZ0214-1*	PIL24012.1 non-ribosomal peptide synthetase (85%)	*Ganoderma sinesnse ZZ0214-1*	Mycocosm + BLASTp + antiSMASH + phylogenetic tree	Figure 2A, Figure S4
†Ganads_822022 Domain1	NA	NA	NA	A0A248AFK6.1 TypeVI siderophore synthetase NPS2 (47%) region: 912 to 1,300	*Gelatoporia subvermispora* B	antiSMASH domain blaspt search, UniProt domain search	Figure S2B
†Ganads_822022 Domain2	NA	NA	NA	A0A248AFK6.1 TypeVI siderophore synthetase NPS2 *Gelatoporia subvermispora* B (46%) region: 1500-1870	*Gelatoporia subvermispora* B	antiSMASH domain blaspt search UniProt domain search	Figure S2B

†Ganads_822022 Domain3	NA	NA	NA	A0A248AFK6.1 TypeVI siderophore synthetase NPS2 *Gelatoporia subvermispora* B (58%) region: 2060-2320	*Gelatoporia subvermispora* B	antiSMASH domain blaspt search UniProt domain search	Figure S2B
*Gands1_ 758095	L-lysine 6-monooxygenase (NADPH-requiring)-domain containing protein	PIL24011.1 hypothetical protein GSI_13762 (92%)	*Ganoderma sinense ZZ0214-1*	KAI1795864.1 L-lysine 6-monooxygenase (NADPH-requiring)-domain-containing protein (88%)	*Ganoderma leucocontextum*	Mycocosm + BLASTp +phylogenetic tree	Figure 1A, Figure S5
*Gands1_700261	Ribosomal protein L24e-domain containing protein	PIL24009.1 hypothetical protein GSI_13760 (96%)	*Ganoderma sinense ZZ0214-1*	TBU35964.1 ribosomal protein L24e-domain-containing protein (96%)	*Dichomitus squalens*	Mycocosm + BLASTp	Figure 1A
*Gands1_884477	Hypothetical protein	PIL24008.1 hypothetical protein GSI_13759 (84%)	*Ganoderma sinense ZZ0214-1*	OJT03542.1 Autophagy-related protein 2 (63%)	*Trametes pubescens*	Mycocosm + BLASTp	Figure 1A
*Gands1_700272	SGNH hydrase	TBU35966.1 SGNH hydrolase-type esterase domain-containing protein (80%)	*Dichomitus squalens*	KAI1795868.1 SGNH hydrolase-type esterase domain-containing protein (86%)	*Ganoderma leucocontextum*	Mycocosm + BLASTp	Figure 1A
*Gands1_ 486561	Transporter	PIL24006.1 transporter (81%)	*Ganoderma sinense ZZ0214-1*	OBZ75358.1 Replication factor A protein 2 (40%)	*Grifola frondosa*	Mycocosm + BLASTp	Figure 1A
*Gands1_776687	Transcription Factor	PIL24005.1 transcription factor (94%)	*Ganoderma sinense ZZ0214-1*	KAI1795871.1 N-acetyltransferase B complex non catalytic subunit-domain-containing protein (91%)	*Ganoderma leucocontextum*	Mycocosm + BLASTp	Figure 1A
*Gands1_517111	Terpene synthase	PIL35630.1 terpene synthase (93%)	*Ganoderma sinense* ZZ0214-1	PIL35630.1 terpene synthase (92%)	Ganoderma sinense ZZ0214-1	Mycocosm + BLASTp + antiSMASH +phylogenetic tree	Figure 1B, Figure S7

*Gands1_690129	Terpene synthase	PIL24516.1 terpene synthase (97%)	*Ganoderma sinense* ZZ0214-1	PIL24516.1 terpene synthase (97%)	*Ganoderma sinense* ZZ0214-1	Mycocosm + BLASTp + antiSMASH + phylogenetic tree	Figure 1B, Figure S7
*Gands1_817038	Terpene synthase	PIL23378.1 terprene synthase (96%)	*Ganoderma sinense* ZZ0214-1	KAI794414.1 terpenoid synthase (89%)	*Ganoderma leucocontextum*	Mycocosm + BLASTp + antiSMASH + phylogenetic tree	Figure 1B, Figure S7

*The MycoCosm top hit corresponds to a protein whose function was computationally predicted based on genome annotation.

†The Alternative Tool top hit corresponds to a protein with experimental characterization.

NA- Analysis did not show a match to one specific organism

Table S3. Detected MS/MS fragment ions for the apo siderophore with MH^+ = 751.4450

m/z	Intensity	*m/z*	Intensity	*m/z*	Intensity
751.4454	4	262.1762	6	184.1333	35
733.4305	5	261.1505	2	182.1172	4
621.3835	2	257.1509	2	178.3974	1
619.3659	2	255.8512	1	172.8707	1
603.3660	2	247.1658	6	166.1228	4
589.0758	2	246.1807	2	164.1060	2
507.3029	36	246.1458	2	157.0609	2
505.0457	2	245.1493	19	149.0921	1
489.2914	13	244.1295	2	148.2612	1
473.2970	12	243.1417	2	144.0798	1
473.0500	2	229.1546	5	133.0972	24
472.6977	2	228.1346	8	131.0816	64
471.2810	8	227.1381	12	131.0692	3
455.2835	5	227.0156	1	130.0860	2
393.2356	2	226.1188	5	118.1737	1
389.0330	3	217.1545	12	116.0705	12
377.2381	9	211.1454	2	115.0866	39
375.2236	11	211.1071	3	115.0763	3
360.2139	2	210.1250	8	114.0551	16
359.2283	38	209.0915	1	113.0708	14
358.1977	4	203.0840	16	113.0611	2
357.2146	6	202.1809	3	86.0601	100
343.2216	3	202.0779	54	85.0761	4
342.2019	8	201.1603	3		
341.2179	17	200.1280	7		
324.1938	2	199.1448	6		
279.1556	4	193.0969	3		
263.1601	27	192.1122	2		

Table S4. Autoconvolution spectrum for the apo siderophore with MH^+ = 751.4450, showing potential neutral losses. All differences between fragment ion masses were calculated and ordered by the number of times each difference between any two MS/MS fragment masses was observed.

m/z	Ocurrence #	Sum formula	Δ*m/z* (mDa)	Unsaturation
114.0681	20	C6H10O2	0.02	2
131.0941	15	C6H13NO2	-0.53	1
244.1425	15	C11H20N2O4	0.19	3
132.0789	14	C6H12O3	0.26	1
96.0568	13	C6H8O	-0.71	3
130.0731	13	C5H10N2O2	-1.13	2
98.0734	12	C6H10O	0.24	2
113.0838	12	C6H11NO	-0.26	2
149.1050	12	C6H15NO3	-0.19	0
226.1306	12	C11H18N2O3	-1.14	4
95.0375	11	C5H5NO	0.39	4
262.1523	11	C11H22N2O5	-0.57	2
80.0621	10	C6H8	-0.5	3
113.0480	10	C5H7NO2	0.32	3
112.0629	10	C5H8N2O	0.63	3
114.0787	10	C5H10N2O	-0.61	2
132.0891	10	C5H12N2O2	-0.78	1
130.0633	9	C6H10O3	0.31	2
97.0533	9	C5H7NO	0.54	3
158.0686	7	C6H10N2O3	-0.54	3
148.0865	6	C5H12N2O3	1.71	1
128.0574	6	C5H8N2O2	-1.18	3
164.0804	5	C5H12N2O4	0.69	1
148.0737	5	C6H12O4	0.14	1
80.0247	5	C5H4O	-1.51	4
111.0326	4	C5H5NO2	0.57	4
96.021	4	C5H4O2	-0.13	4
86.0834	4	C4H10N2	-1	1

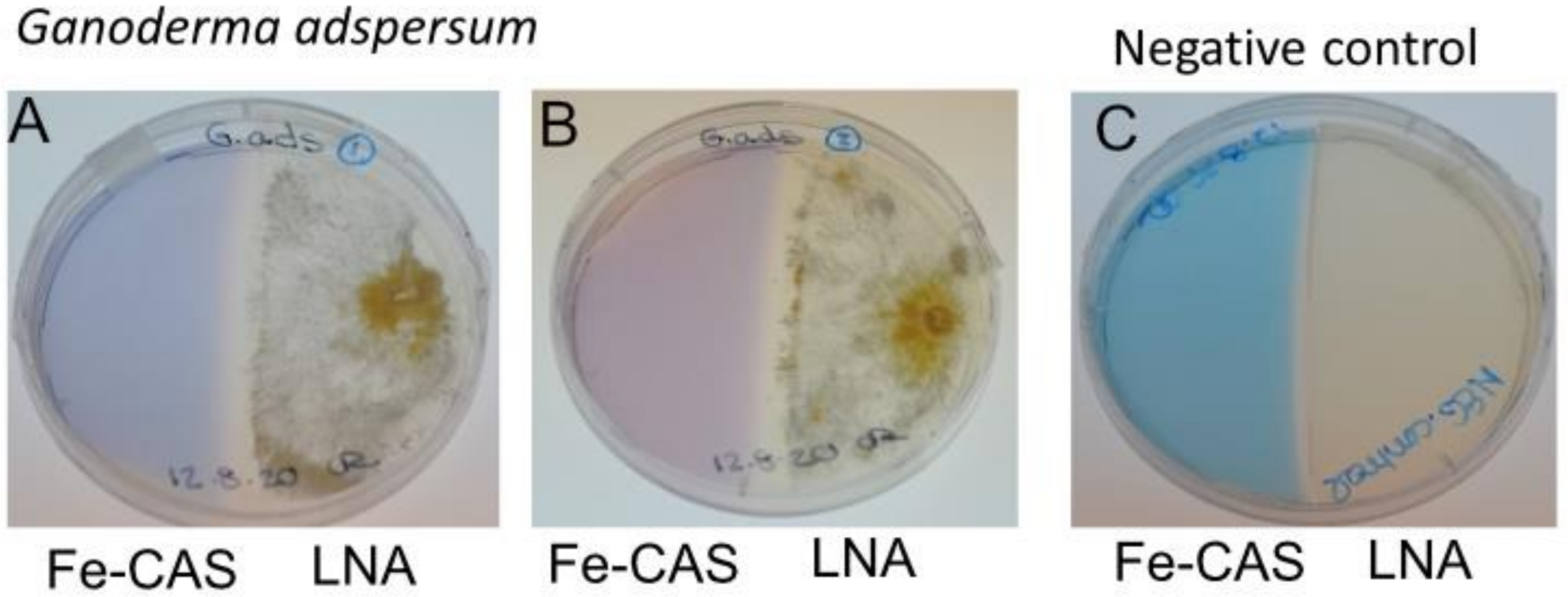

Figure S1. Growth and siderophore secretion by *G. adspesrum* under iron-limited conditions. LNA Petri dishes containing Fe-CAS reagent or LNA only, were used to detect siderophore secretion. **A and B**) A purple violet color indicates siderophores were secreted into the Fe-CAS side of the Petri-dish by *G.adspersum* growing under Fe limited conditions on the LNA side of the Petri dish*.* **C)** A control Petri dish with no fungus. See the main text for Methods and Results.

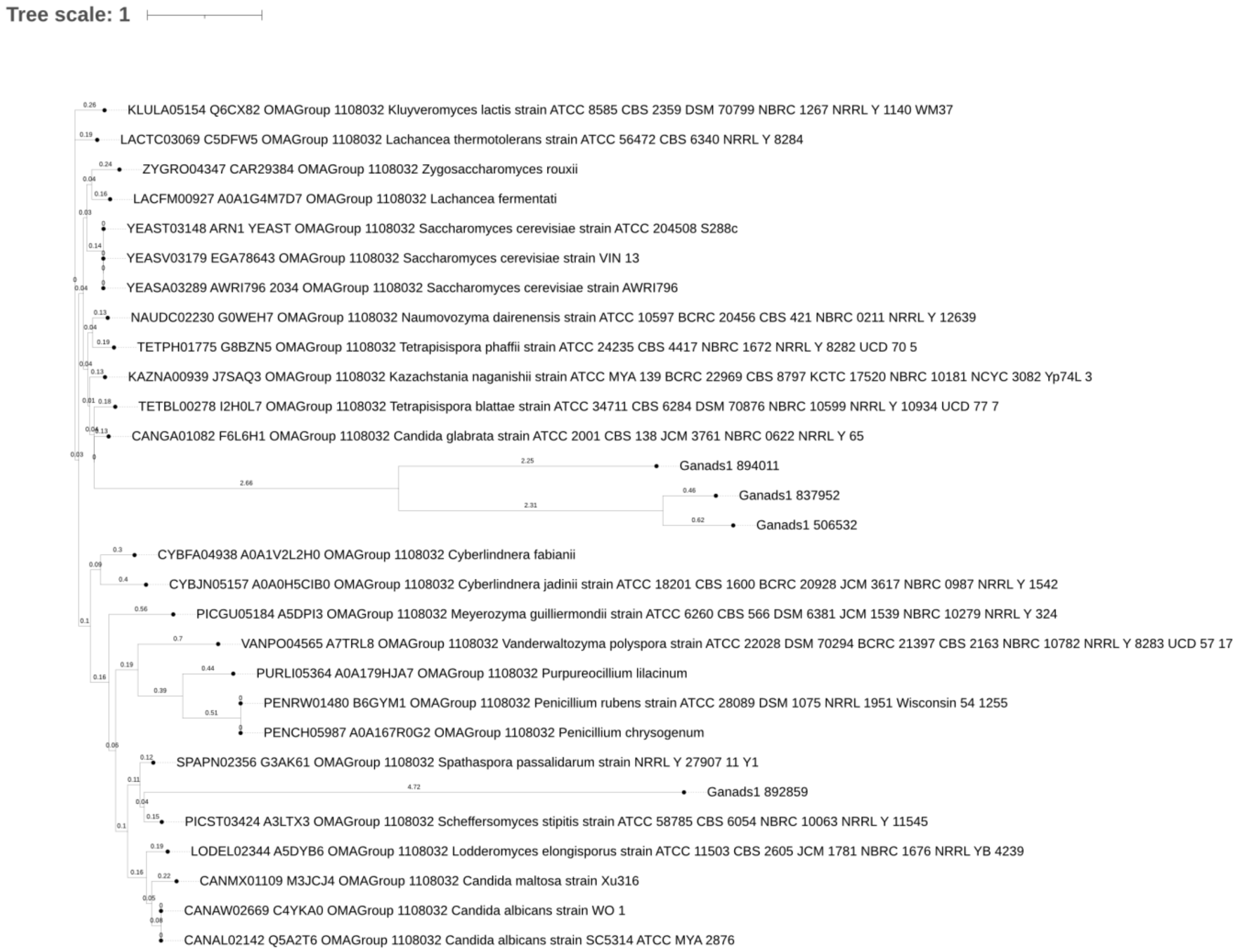

Figure S2. Phylogenetic tree of *G. adspersum* transporter sequences and orthologous sequences of fungal Major Facilitator Superfamily (MFS) proteins. *G. adsperum* sequences are labeled Gands1. The tree was constructed using NGPhylogeny.fr web server (https://ngphylogeny.fr) with the "One Click" workflow. Multiple sequence alignment was performed using MAFFT, alignment curation with BMGE, and phylogenetic inference with PhyML. Branch support values represent approximate likelihood ratio test (aLRT) scores calculated by PhyML and are shown as nodes.

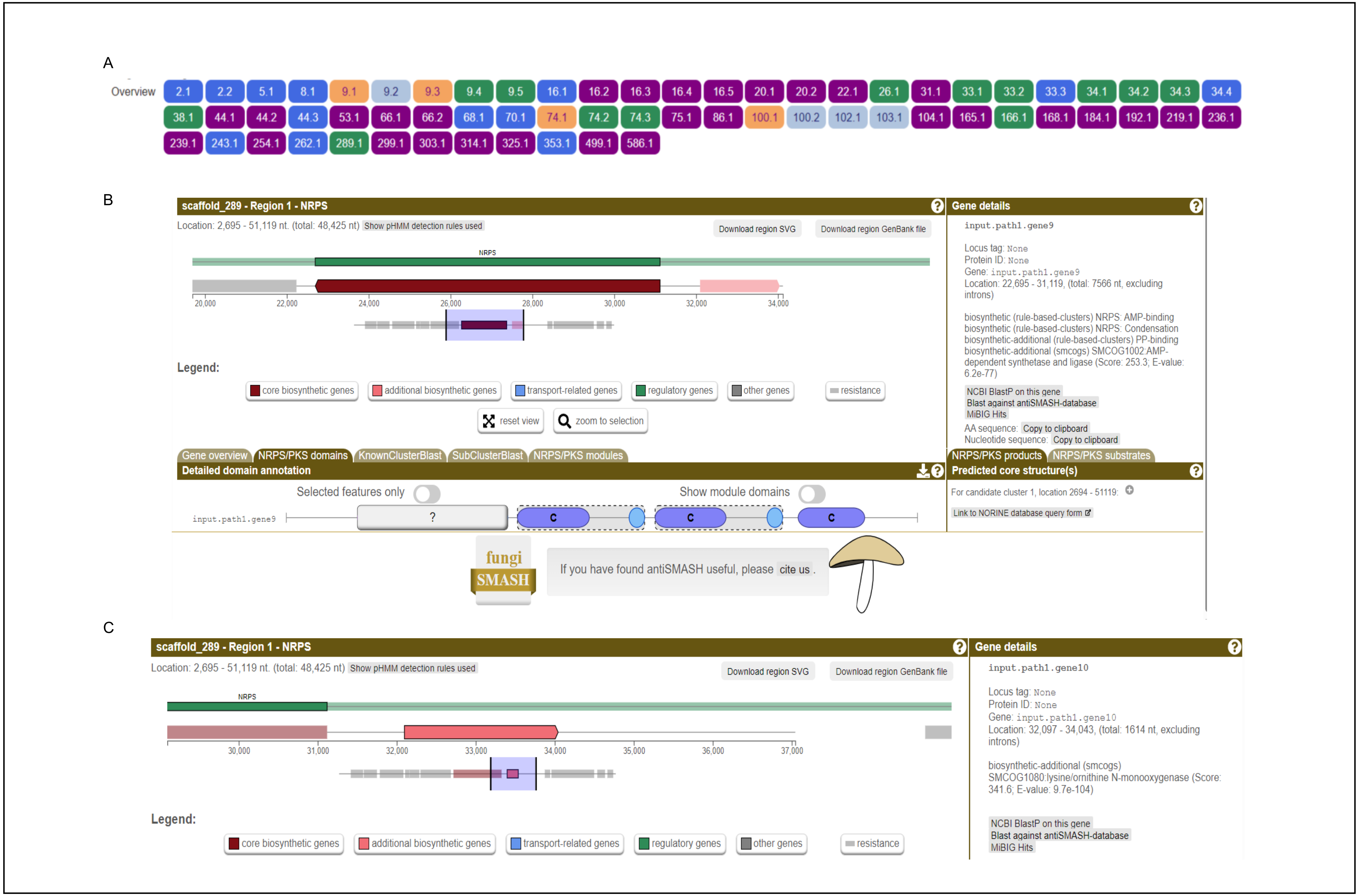
A
Overview
2.1 2.2 5.1 8.1 9.1 9.2 9.3 9.4 9.5 16.1 16.2 16.3 16.4 16.5 20.1 20.2 22.1 26.1 31.1 33.1 33.2 33.3 34.1 34.2 34.3 34.4
38.1 44.1 44.2 44.3 53.1 66.1 66.2 68.1 70.1 74.1 74.2 74.3 75.1 86.1 100.1 100.2 102.1 103.1 104.1 165.1 166.1 168.1 184.1 192.1 219.1 236.1
239.1 243.1 254.1 262.1 289.1 299.1 303.1 314.1 325.1 353.1 499.1 586.1
B
scaffold_289 - Region 1 - NRPS
Location: 2,695 - 51,119 nt. (total: 48,425 nt) Show pHMM detection rules used
Download region SVG
Download region GenBank file
NRPS
20,000 22,000 24,000 26,000 28,000 30,000 32,000 34,000
Legend:
core biosynthetic genes
additional biosynthetic genes
transport-related genes
regulatory genes
other genes
resistance
reset view
zoom to selection
Gene details
input.path1.gene9
Locus tag: None
Protein ID: None
Gene: input.path1.gene9
Location: 22,695 - 31,119, (total: 7566 nt, excluding introns)
biosynthetic (rule-based-clusters) NRPS: AMP-binding
biosynthetic (rule-based-clusters) NRPS: Condensation
biosynthetic-additional (rule-based-clusters) PP-binding
biosynthetic-additional (smcogs) SMCOG1002:AMP-dependent synthetase and ligase (Score: 253.3; E-value: 6.2e-77)
NCBI BlastP on this gene
Blast against antiSMASH-database
MiBIG Hits
AA sequence: Copy to clipboard
Nucleotide sequence: Copy to clipboard
Gene overview
NRPS/PKS domains
KnownClusterBlast
SubClusterBlast
NRPS/PKS modules
Detailed domain annotation
Selected features only
Show module domains
input.path1.gene9
?
C
C
C
NRPS/PKS products
NRPS/PKS substrates
Predicted core structure(s)
For candidate cluster 1, location 2694 - 51119:
Link to NORINE database query form
fungi SMASH
If you have found antiSMASH useful, please cite us .
C
scaffold_289 - Region 1 - NRPS
Location: 2,695 - 51,119 nt. (total: 48,425 nt) Show pHMM detection rules used
Download region SVG
Download region GenBank file
NRPS
30,000 31,000 32,000 33,000 34,000 35,000 36,000 37,000
Legend:
core biosynthetic genes
additional biosynthetic genes
transport-related genes
regulatory genes
other genes
resistance
Gene details
input.path1.gene10
Locus tag: None
Protein ID: None
Gene: input.path1.gene10
Location: 32,097 - 34,043, (total: 1614 nt, excluding introns)
biosynthetic-additional (smcogs) SMCOG1080:lysine/ornithine N-monooxygenase (Score: 341.6; E-value: 9.7e-104)
NCBI BlastP on this gene
Blast against antiSMASH-database
MiBIG Hits

Figure S3. **antiSMASH output showing predicted biosynthetic gene clusters in the *Ganoderma adspersum* genome. A).** Overview of all scaffolds in the *G. adspesrum* genome annotated by antiSMASH. The numbers indicate scaffold and cluster number. The colors of the boxes represent different biosynthetic functions assigned to those clusters. Red indicates core biosynthetic genes that encode the main enzymes for natural product synthesis. Orange indicates additional biosynthetic genes. Blue indicates transport related genes involved in the transport of metabolites across membranes. Green indicates regulatory genes including transcription factors or other regulators of gene expression. Purple indicates other genes that are non-biosynthetic, unclassified or accessory genes. Gray indicates hypothetical or poorly characterized genes. **B).** Detailed views of the identified NRPS cluster, showing core biosynthetic (red), additional biosynthetic (pink), transport-related (blue), regulatory (green), and other (blue) genes. Domain architecture of the NRPS gene includes adenylation (A), thiolation (T), and condensation (C) domains, consistent with siderophore-type assembly lines. **C).** Detailed view of the additional biosynthethic gene right next to the identified NRPS gene identified as a monooxygenase. Annotations and predictions were generated using the antiSMASH pipeline

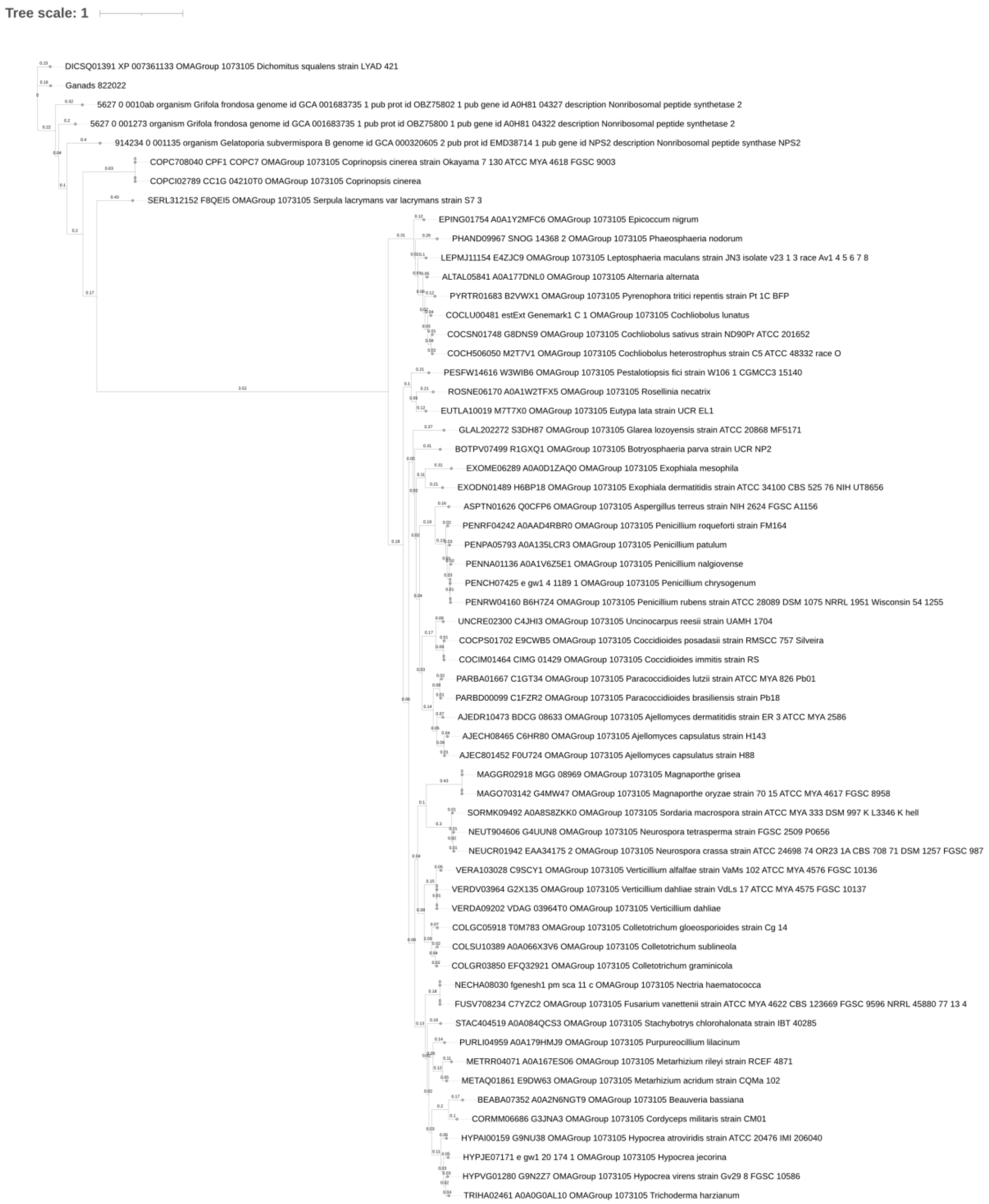

Figure S4. Phylogenetic tree of *G. adspersum* Gands_822022 and orthologous sequences of fungal NRPS proteins. *G. adsperum* sequence is labeled Gands1 822022.The tree was constructed using NGPhylogeny.fr web server (https://ngphylogeny.fr) with the "One Click" workflow. Multiple sequence alignment was performed using MAFFT, alignment curation with BMGE, and phylogenetic inference with PhyML. Branch support values represent approximate likelihood ratio test (aLRT) scores calculated by PhyML and are shown as nodes.

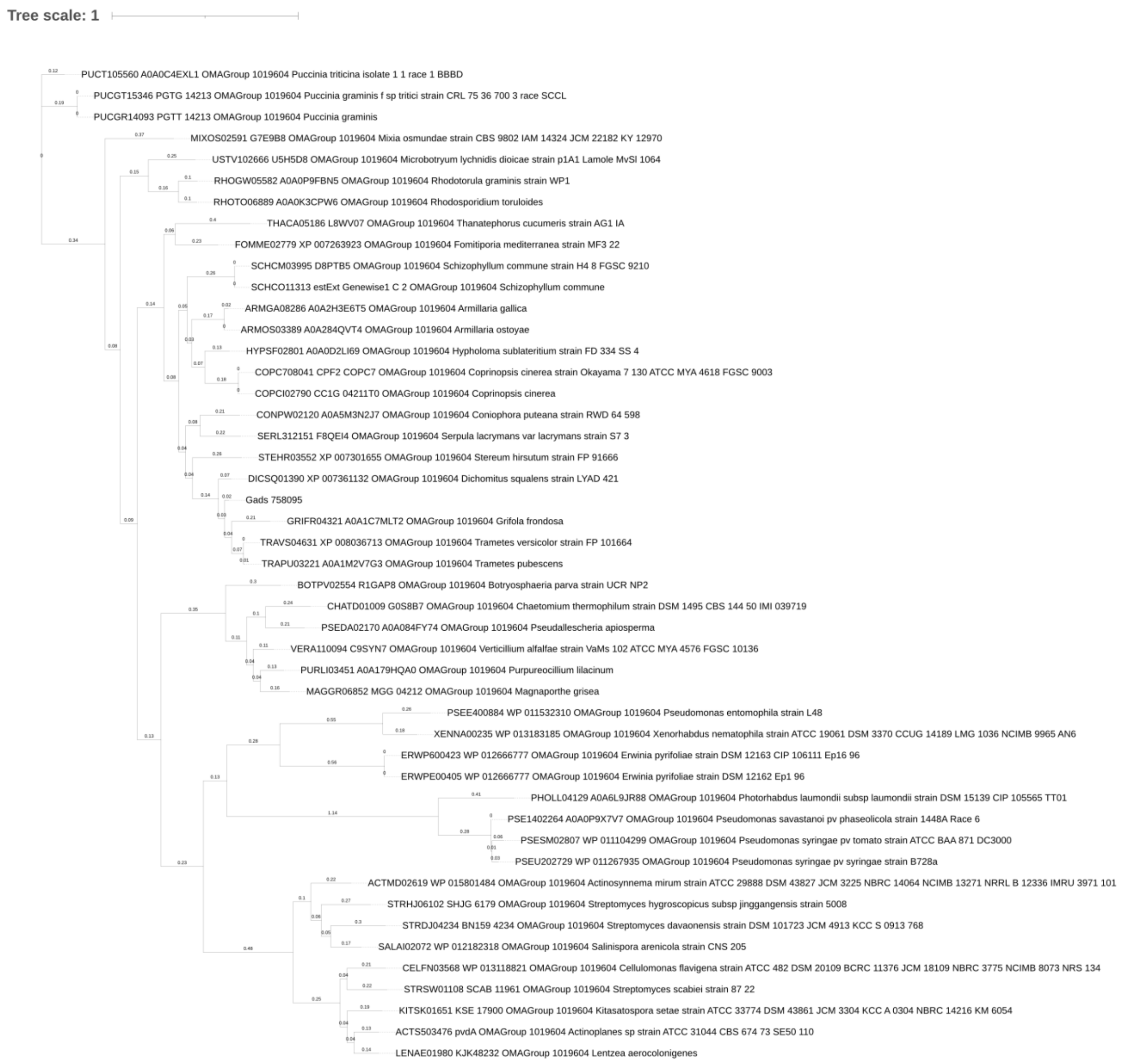

Figure S5. Phylogenetic tree of *G. adspersum* Gands_758095 and orthologous sequences of fungal NRPS proteins. *G. adsperum* sequence is labeled Gands1 758095. The tree was constructed using NGPhylogeny.fr web server (https://ngphylogeny.fr) with the "One Click" workflow. Multiple sequence alignment was performed using MAFFT, alignment curation with BMGE, and phylogenetic inference with PhyML. Branch support values represent approximate likelihood ratio test (aLRT) scores calculated by PhyML and are shown as nodes.

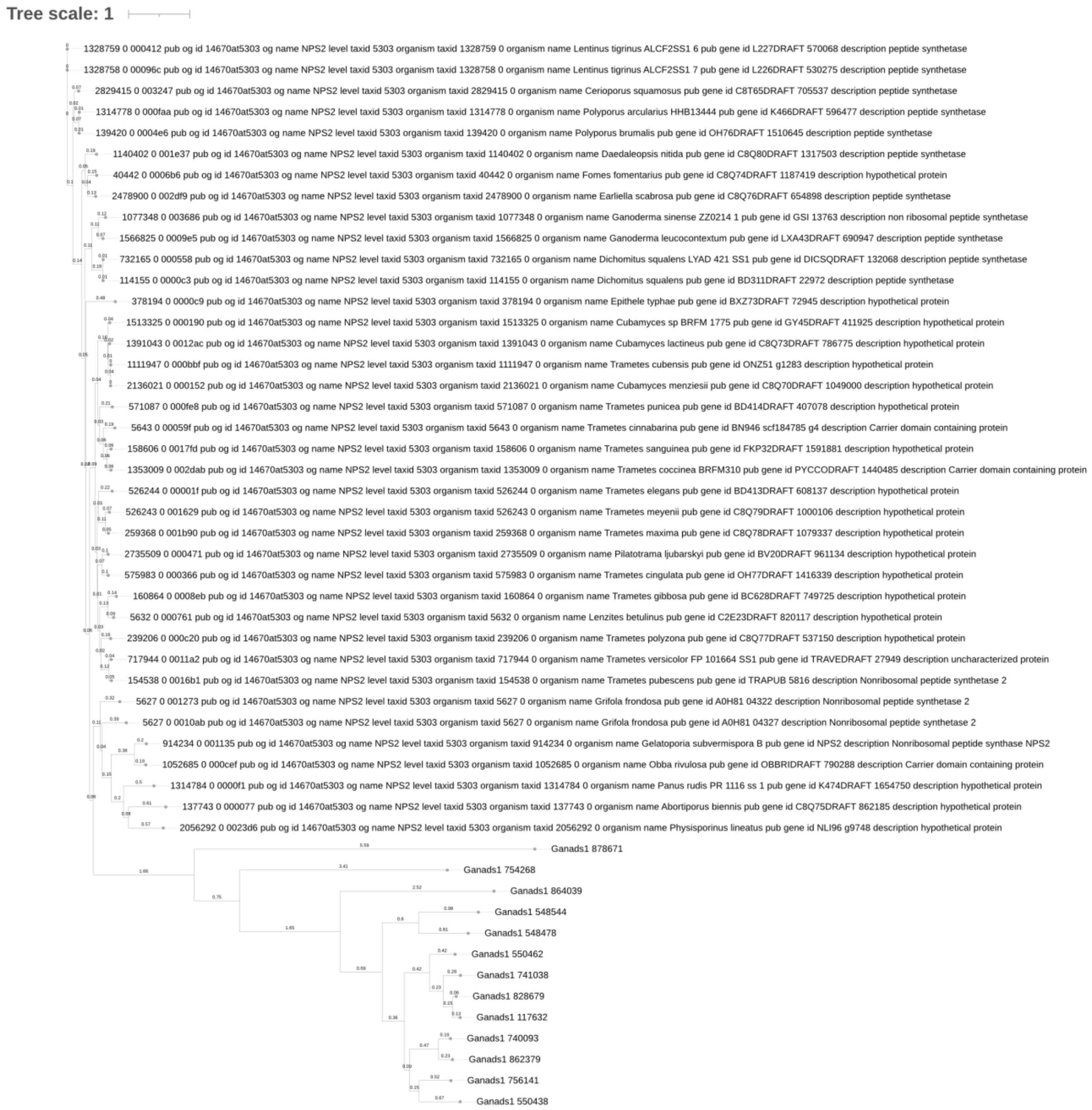

Figure S6. Phylogenetic tree of *G. adspersum* NRPS-like sequences and orthologous sequences of fungal NRPS proteins. *G. adsperum* sequences are labeled Gands1. The tree was constructed using NGPhylogeny.fr web server (https://ngphylogeny.fr) with the "One Click" workflow. Multiple sequence alignment was performed using MAFFT, alignment curation with BMGE, and phylogenetic inference with PhyML. Branch support values represent approximate likelihood ratio test (aLRT) scores calculated by PhyML and are shown as nodes.

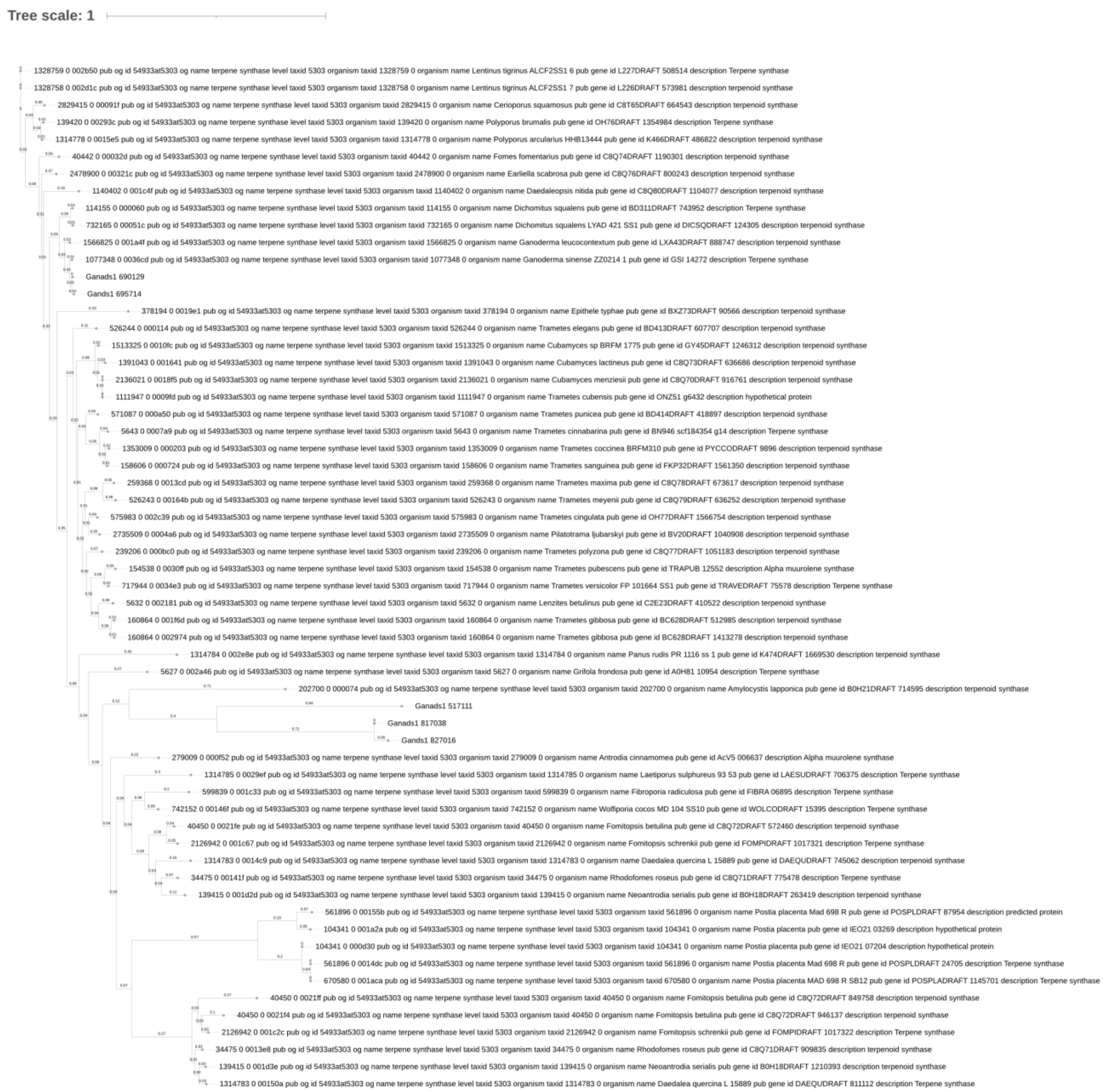

Figure S7. Phylogenetic tree of *G. adspersum* terpene sequences and orthologous sequences of fungal terpene synthase proteins. *G. adsperum* sequences are labeled Gands1. The tree was constructed using NGPhylogeny.fr web server (https://ngphylogeny.fr) with the "One Click" workflow. Multiple sequence alignment was performed using MAFFT, alignment curation with BMGE, and phylogenetic inference with PhyML. Branch support values represent approximate likelihood ratio test (aLRT) scores calculated by PhyML and are shown as nodes.

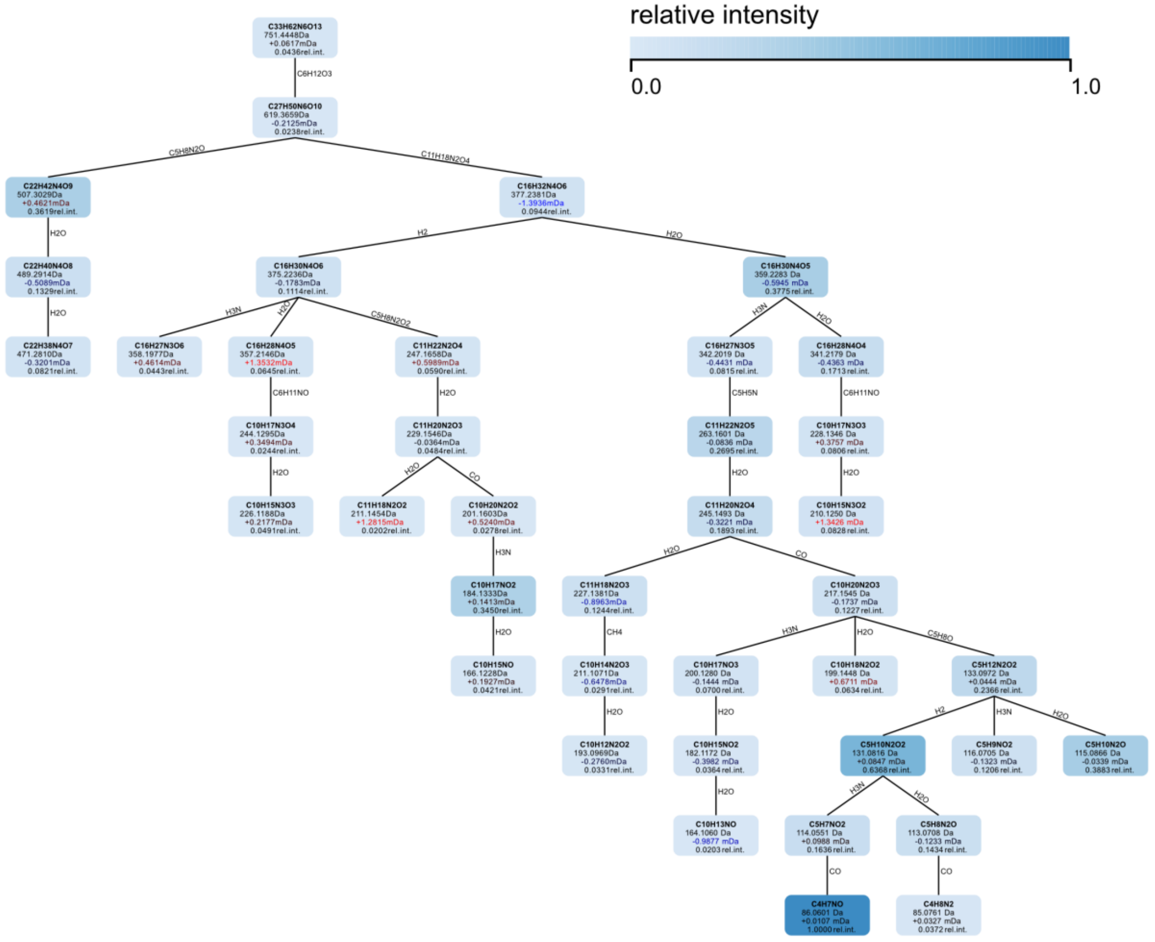

Figure S8. MS/MS fragmentation tree generated with the software tools SIRIUS and CSI:FingerID (1, 2).

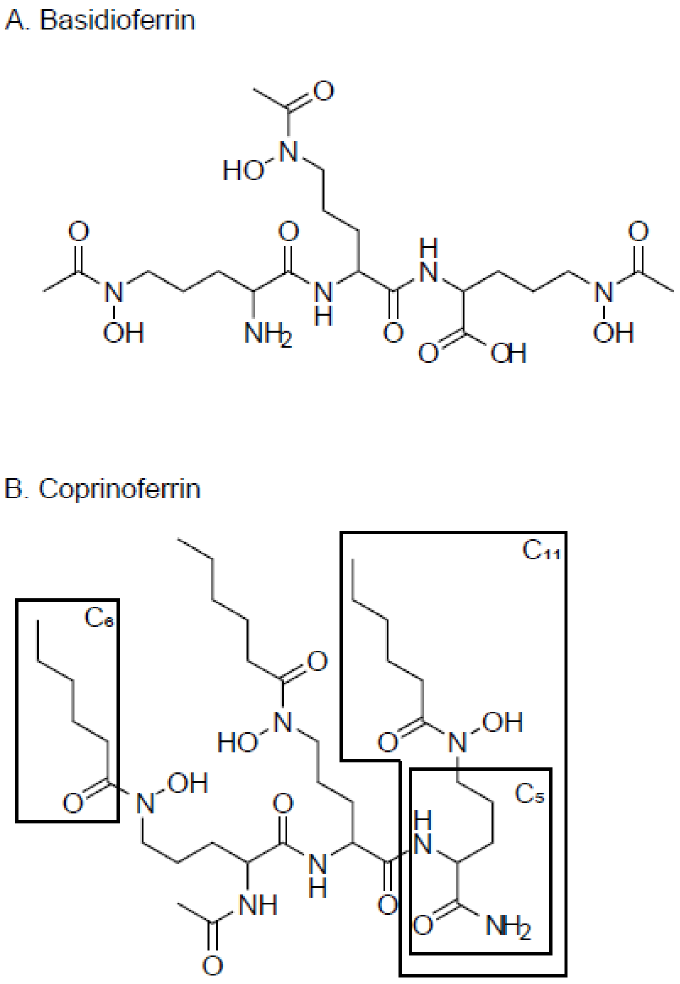

Figure S9. Structures of (A) Basidioferrin and (B) Coprinoferrin. Potential fragments containing C6, 11, and C5 are indicated which were in agreement with fragment and neutral loss observations for the new siderophore in this study (Figure S8).

Supporting References

1. Böcker, Sebastian, Letzel MC, Lipták Z, Pervukhin A. 2009. SIRIUS: decomposing isotope patterns for metabolite identification. Bioinformatics 25:218–224.

2. Dührkop K, Shen H, Meusel M, Rousu J, Böcker S. 2015. Searching molecular structure databases with tandem mass spectra using CSI:FingerID. Proc Natl Acad Sci USA 112:12580–12585.
